# Presenilin-1 F105C mutation leads to tau accumulation in human neurons via the Akt/mTORC1 signaling pathway

**DOI:** 10.1186/s13578-022-00874-8

**Published:** 2022-08-14

**Authors:** Cheong-Meng Chong, Yuan Tan, Jiaqi Tong, Minjing Ke, Ke Zhang, Lingli Yan, Xiaotong Cen, Jia-Hong Lu, Guobing Chen, Huanxing Su, Dajiang Qin

**Affiliations:** 1 State Key Laboratory of Quality Research in Chinese Medicine, Institute of Chinese Medical Sciences, University of Macau, Macao, China; 2 Key Laboratory of Biological Targeting Diagnosis, Therapy and Rehabilitation of Guangdong Higher Education Institutes, The Fifth Affiliated Hospital of Guangzhou Medical University, Guangzhou, China; 3 Institute of Geriatric Immunology, School of Medicine, Jinan University, 510623 Guangzhou, People’s Republic of China

**Keywords:** mTOR, Presenilin-1, iPSC, CRISPR/Cas9

## Abstract

**Background:**

The mammalian target of rapamycin (mTOR) plays a critical role in controlling cellular homeostasis, and its dysregulation has been implicated in Alzheimer’s disease (AD). Presenilin-1 (PS1) mutations account for the most common causes of familial Alzheimer’s disease (FAD); however, whether PS1 mutation causes mTOR dysregulation in human neurons remains a key unresolved issue.

**Methods:**

We generated heterozygotes and homozygotes of PS1 F105C knock-in mutation in human induced pluripotent stem cells (iPSCs) via CRISPR/Cas9/piggyback-based gene editing and differentiated them into human neurons. Secreted Aβ and tau accumulation were determined by ELISA assay, immunofluorescence staining, and western blotting analysis. mTOR signaling was evaluated by western blotting analysis, immunofluorescence staining, and co-immunoprecipitation. Autophagy/lysosome activities were determined by LC3-based assay, LysoTracker Red staining, and DQ-Red BSA staining.

**Results:**

Through comparison among these isogenic neurons, PS1 F105C mutant neurons exhibited elevated Aβ and tau accumulation. In addition, we found that the response of mTORC1 to starvation decreases in PS1 F105C mutant neurons. The Akt/mTORC1/p70S6K signaling pathway remained active upon EBSS starvation, leading to the co-localization of the vast majority of mTOR with lysosomes. Consistently, PS1 F105C neurons displayed a significant decline in starvation-induced autophagy. Notably, Torin1, a mTOR inhibitor, could efficiently reduce prominent tau pathology that occurred in PS1 F105C neurons.

**Conclusion:**

We demonstrate that Chinese PS1 F105C mutation causes dysregulation of mTORC1 signaling, contributing to tau accumulation in human neurons. This study on inherited FAD PS1 mutation provides unprecedented insights into our understanding of the molecular mechanisms of AD. It supports that pharmaceutical blocking of mTOR is a promising therapeutic strategy for the treatment of AD.

**Supplementary Information:**

The online version contains supplementary material available at 10.1186/s13578-022-00874-8.

## Introduction

Alzheimer’s disease (AD) is the most common neurodegenerative disorder characterized by progressive memory loss and cognitive impairment. Mutations in the *PSEN1* gene, encoding presenilin-1 (PS1), account for the highest number of cases of familial AD (FAD) [1]. So far, over 300 FAD PS1 mutations have been reported, which leads to the manifestation of AD-associated clinical and pathological features such as amyloid-β (Aβ) plaques, tau neurofibrillary tangles, synaptic abnormalities, and neuronal loss [2–7]. Despite extensive studies on the pathogenesis of PS1 mutations, the exact mechanisms are still being highly debated. Many investigations using overexpression of PS1 mutants or fibroblasts from FAD PS1 mutant patients have shown that γ-secretase-mediated alteration of amyloid precursor protein (APP) processing may be accompanied or even preceded by γ-secretase independent impairments such as the dysfunctions in the endolysosomal and the autophagic system [8–12]. Human neurons with the PS1^ΔE9^ mutation exhibited defective endolysosomal trafficking and decreased transcytosis from soma to axon [13]. The pathogenic PS1 mutations Y115H, L166P, C410Y, or L435F, were reported to exert loss-of-function on the activity of Aβ production, whereas the pathogenic PS1 S365A mutation was reported to increase Aβ production [14]. Collectively, these studies on inherited FAD PS1 mutation provide unprecedented insights into our understanding of the molecular mechanisms of AD.

The mechanistic target of rapamycin (mTOR) is a serine/threonine protein kinase, known to control cellular homeostasis [15]. In mammals, mTOR is the core subunit of mTOR complex 1 (mTORC1) and mTORC2. These complexes regulate their downstream substrates via mTOR kinase activity. Tau pathology is caused by the aggregation of abnormally hyperphosphorylated tau protein in the soma and partial neurite of neurons [16–20]. In the AD brain, neurons do not appear to remove this kind of tau aggregation. In general, intracellular homeostasis systems such as autophagy can clear insoluble tau [21–24]. Given a critical role in regulating protein homeostasis in response to various stresses, insensitive mTORC1 may be implicated in the pathological protein accumulations of AD. Although increasing pieces of evidence support that PS1 mutations lead to tau pathology and autophagy dysfunction in human neurons [25–28], whether PS1 mutation causes mTORC1 dysregulation in human neurons remains a key unresolved issue.

Human induced pluripotent stem cells (iPSCs) hold considerable promise for modeling human disease development because they possess the genetic predispositions of diseases of interest. Human iPSC avoids species differences and more precisely recapitulates human AD development. Various AD phenotypes can be observed in human iPSCs-derived neurons with FAD PS1 mutations [29–31]. However, individual genomic diversity restricts the application of this model in investigating the pathogenic mechanisms of PS1 mutations. Targeted genome editing to introduce precise mutations into human iPSCs is a rigorous and encouraging strategy for generating human disease models with the same genetic background. Although the ability of iPSC-based models to mimic AD has a limitation, in part due to the loss of age-dependent cellular phenotypes, it has been proved to be useful for exploring the exact mechanisms of PS1 mutants and providing reasonable evidence to link to human AD neuronal characteristics [13, 32].

**Fig. 1:**
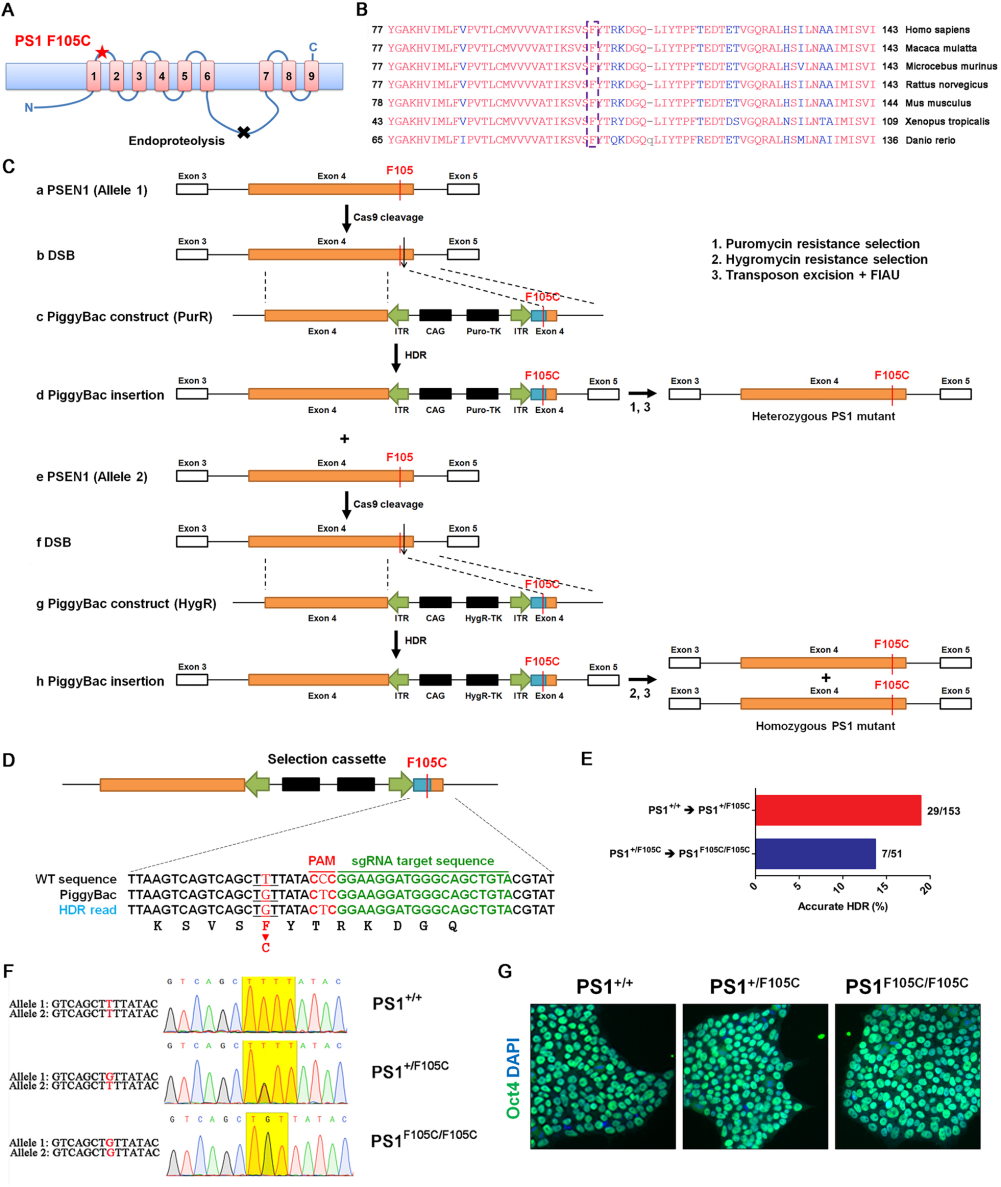
Seamless introduction of heterozygous and homozygous PS1 F105C knock-in mutation into healthy human iPSCs using a combination of CRISPR/Cas9 system and piggyBac transposon. **A** Schematic representation of the PS1 F105C protein. Asterisk represents the mutation site in the transmembrane domain 1 of PS1. Cross represents the endoproteolysis cleavage site of PS1. **B** Sequence alignment and conservation of F105 residue in PS1 of different species. **C** Strategy for seamless introduction of the heterozygous F105C mutation **a**–**d** and the homozygous F105C mutation (**a**–**h**). **a** Location of F105 in the exon 4 of PSEN1 allele 1; **b** DSB in the exon 4 of allele 1 after Cas9 cleavage; **c** Targeting construct of the piggyBac transposon carrying the selectable markers puro-TK, flanked by 500 bp of PS1 wild-type genomic sequences; **d** Insertion of the piggyBac transposons through HDR; **e** Location of F105 in the exon 4 of PSEN1 allele 2; **f** DSB in the exon 4 of allele 2 after Cas9 cleavage; **g** The targeting construct of the piggyBac transposon carrying the selectable markers HygR-TK, flanked by 500 bp of PS1 wild-type genomic sequences. **d** Insertion of the other piggyBac transposons through HDR. After **a**–**d**, the cells were selected and amplified in a medium containing puromycin to generate the heterozygous PS1 F105C mutant. Puromycin-resistant clones with heterozygous PS1 F105C were transfected with transposase expression plasmids and treated with FIAU to remove the piggyBac-containing clones. Second gene editing was performed in the puromycin-resistant clones with desired heterozygous PS1 F105C and piggyBac to generate the homozygous PS1 F105C mutant. After e–h, the cells were selected and amplified in a medium containing hygromycin. Hygromycin-resistant clones with the desired homozygous PS1 F105C were then transfected with transposase expression plasmids and treated with FIAU to remove piggyBac-containing clones for both alleles. *CAG* CAG promoter, *ITR* inverted terminal repeats. **D** PSEN1 sequencing alignment showing Cas9 cleavage site and intended mutation site for modifying F105C. **E** The percentage of accurate HDR using this combined strategy. **F** Sanger sequencing was used to identify PS1 with F105C mutation. **G** Oct4 immunostaining of iPSC lines

The F105 residue is located between the transmembrane domains 1 and 2 of PS1 (Fig. 1A), which is highly conserved in various species (Fig. 1B). PS1 F105C, F105I, F105L, and F105V mutations were reported to cause FAD, suggesting that the mutations in the F105 residue have a high risk of being pathogenic. F105C mutation is a missense mutation which is first identified in the early-onset FAD Chinese patients [33]. However, the pathogenic mechanisms of PS1 F105C mutation lead to neurodegeneration maintains unknown. In this study, we, for the first time, generated heterozygous and homozygous PS1 F105C mutant iPSCs using CRISPR/Cas9 and PiggyBac transposon. This model is used for investigating the impacts of PS1 F105C mutation with and without wild-type PS1 interference on mTOR signaling in human neurons. Our results demonstrate that the PS1 F105C mutation causes dysregulation of Akt/mTORC1 signaling, contributing to tau accumulation in human neurons.

## Materials and methods

### Human UC-H2-iPSC generation and characterization

We reprogrammed urine cells according to a previously reported procedure [34]. In brief, urine cells were collected from a 28-year-old healthy female Chinese donor with informed consent. We explained the purpose and process for collecting urine cells and generating iPSCs to the donor in great detail. To collect the exfoliated cells, ~ 500 ml of a mid-stream urine sample was obtained from the donor and centrifuged. The primary urine cells were cultured in a urine cell medium comprising DMEM/F12 medium (Gibco, Grand Island, NY, USA) supplemented with 10% fetal bovine serum, 1 mM GlutaMAX (Life Technologies, Carlsbad, CA, USA), 0.1 mM non-essential amino acids, 0.1 mM β-mercaptoethanol, and a SingleQuot Kit (CC-4127 REGM; Lonza, Portsmouth, NH, USA). After amplifying the urine cells, a pCEP4 episomal vector containing the SOX2, OCT4, KLF4, and SV40LT genes [35] and the other pCEP4 vector carrying a miR302–367 precursor [36], were co-transfected into urine cells by nucleofection (Amaxa Basic Nucleofector Kit for primary mammalian epithelial cells, T-013 program; Lonza). The transfected urine cells were plated on Matrigel (BD Biosciences, San Diego, CA, USA)-coated six-well plates (1–3 × 10^5^ cells per well) in a urine cell culture medium for the first two days. Then the media was changed to mTeSR1 (STEMCELL Technologies, Cambridge, MA, USA). The medium was exchanged every two days until 15 days after transfection, and then colonies were collected and transferred onto a new Matrigel plate using mTeSR1. The cells were dissociated into small clusters or single cells for further expansion. Compared to the H9 human embryonic stem cell line, we analyzed the expression of pluripotency genes by quantitative reverse transcription PCR (qRT-PCR) analysis. The expression of the pluripotent marker Nanog was confirmed by immunofluorescence. We performed karyotype analysis to identify whether UC-H2-iPSC cells had a normal karyotype. We used the teratomas formation assay to evaluate the pluripotency.

### Karyotype analysis

When iPSCs covered 80% of 6-cm plates, we added demecolcine up to a concentration of 0.2 µg/ml for 2 h. The cells were then trypsinized, collected by centrifugation, re-suspended in 7 ml of 0.075 M KCl solution, and incubated at 37 °C for 25 min. A fixative solution (7 ml) composed of 75% methanol and 25% acetic acid was added to the solution, mixed gently, and incubated for 5 min at 37 °C. The supernatant was then removed after centrifugation, and then a fixative solution (7 ml) was added to the cell pellets and gently mixed. The cells were then dropped on a cold slide and incubated at 75 °C for 3 h. The metaphase spreads prepared from cells treated with trypsin and Giemsa staining were analyzed using an Olympus BX51 microscope.

### Construction of the piggybac-based donor plasmid

To knock in the F105C mutation in the exon of the PS1 gene, we modified the homologous arms of the piggyBac plasmid (pTK-puroDTK-Neo, a gift from Dr. Yuet Wai Kan) to construct the piggyBac donor plasmids according to a previously described protocol [37]. We used a PCR method to connect the 5′ homologous and 3′ homologous arms to the piggyBac cassette. The homologous arms were then amplified using UC-H2-iPSC as the template. Finally, the Neo resistance gene was replaced with either the Puro or HygR gene to generate two types of piggyBac-based donors.

### Gene targeting and removal of the piggyBac transposon cassette

For gene targeting, approximately 3 × 10^6^ iPSCs were transferred to a Matrigel-coated culture dish and grown in mTeSR1™ containing 10 µM of Y27632 (Calbiochem, USA). On the second day, the cells were transfected with a mixture of Cas9, gRNA, and the donor plasmid containing Puro (30 µg) using lipofectamine under the guidance of the Lipofectamine™ 3000 reagent protocol. On the third day, the cells were cultured in mTeSR1™ containing 10 µM Y27632 for the entire day. The puromycin selection (2 mg/mL) started on day 3 after transfection and continued for five days. The resistant colonies were identified and expanded for PCR screening. All primers are listed in Additional file 1: Tables S1 and S2.

To remove the piggyBac cassette, 3 × 10^6^ iPSCs were transfected with 90 µg hyperactive transposase vector and hyperPBase (a gift from Dr. Yuet Wai Kan and Dr. Lin Ye) via lipofection, followed by selection with FIAU (0.25 mM) for six days. To detect the excision of the piggyBac cassette, we used P1, P2, puro-seq-F, puro-seq-R, HygR-F, and HygR-R to detect the removal of the PB transposon cassette from the genome. All primers are listed in Additional file 1: Tables S1 and S2.

### Single-cell derived iPSC clonal analysis

The cells were plated on Matrigel-coated 96-well culture plates at a density of 0.5 cells/well in 96-well plates to ensure that the cell clone was derived from a single cell and grown in mTeSR1™ containing 10 µM Y27632 for eight days after the cell expansion culture.

### DNA extraction and mutation analysis

We used an animal tissue direct PCR lit (Foregene) to extract and amplify the target genomic DNA of cell clones from 96-well plates. To identify the mutation site sequence in the genome, we used a pair of primers (P1 and P2) to amplify the sequence, including the exon 4 of the PS1 gene. To identify piggyBac integration via homologous recombination, the primers devoted to junction PCR were designed as follows. P3 was selected to lie on the homologous region of the 3′ arm, and P4 was designed within the piggyBac cassette. These two primers were used to amplify the 750 bp product. The P5 and P6 primers were used to amplify the 805 bp product between the homologous 5′ arm and the piggyBac cassette. Only the target sites in the cells integrated with the piggyBac cassette could be amplified.

### Detection of off-target

Both heterozygote and homozygote F105C mutants were grown in six-well plates to prepare the genomic DNA (gDNA), which was isolated using the Wizard® Genomic DNA Purification Kit (Promega, Madison, WI, USA) for off-target detection. Each gRNA was searched using the COSMID tool [38] (http://crispr.bme.gatech.edu) to evaluate the potential for off-target gRNA. We confirmed all the sites amplified by PCR by sequencing. The primers and off-target analytical results are shown in Additional file 1: Tables S3 and S4, respectively. To detect the residual piggyBac cassettes, we amplified the key elements Puro and HygR after selection with FIAU. These two key elements could be amplified only when the cells were integrated with piggyBac cassettes.

### NSCs induction and culture

We generated NSCs from iPSCs as described previously [39]. In brief, iPSCs were split as cell clumps and plated on six-well tissue culture plates coated with Matrigel in PSC Neural Induction Medium (Life Technologies) with 10 µM Y27632 at a density of 2.5–3.0 × 10^4^ cells/cm^2^. The Neural Induction Medium (NIM) was changed every other day from day 1 to day 5. After day 5, the NIM was changed every day until day 7. The cells were then dissociated on day 7 using accutase, re-suspended in the NIM containing 10 µM Y27632, and plated on Matrigel-coated dishes at 1.2 × 10^5^ cells/cm^2^. The NIM was changed every other day until the NSCs were 90% confluent. The NSCs were cryopreserved in NIM containing 10% dimethyl sulfoxide (DMSO) (Sigma-Aldrich, St. Louis, MO, USA).

### Neuron differentiation

NSCs at passage 6 were digested with accutase to single cells. Then the cells were seeded on poly-L-ornithine/laminin-coated plates at a density ranging from 5 × 10^4^ to 10 × 10^4^ cells/cm^2^ for 20 days of culturing in N2B27 medium (DMEM-F12/Neurobasal medium 1:1 with 2% B27, 1% N2, 1% non-essential amino acids, and 2 mM Glutamax) (all Life Technologies) supplemented with GDNF (PeproTech, 20 ng/mL), BDNF (PeproTech, 20 ng/mL), NT3 (PeproTech, 10 ng/mL), ascorbic acid (Sigma Aldrich, 200 µM), and cAMP (Sigma Aldrich, 10 µM).

### Quantitative reverse transcription PCR analysis

Total RNA was isolated using the RNA-Solv Reagent (OMEGA). We performed reverse transcription with RNA (2 µg) using ReverTra Ace (TOYOBO) and Oligo(dT)18 (TaKaRa). qRT-PCR was performed on a SYBR® Premix Ex Taq (TaKaRa) using ViiA™ 7 Real-Time PCR System (Thermo Fisher Scientific). The reaction procedures are as follows: an initial step at 95 °C for 5 min, 40 cycles of 94 °C for 15 s, and 60 °C for 34 s. All gene primers are listed in Additional file 1: Table S5.

### Immunofluorescence staining

The cells for immunostaining were fixed in 4% paraformaldehyde at room temperature for 20 min, blocked with 5% donkey serum in 0.3% Triton™ X-100 for 1 h, and then incubated with primary antibodies overnight at 4 °C. After washing three times with Dulbecco’s phosphate-buffered saline (DPBS), the cells were incubated at room temperature for 1 h with fluorophore-conjugated secondary antibodies (Life Technologies). After immunostaining, the cells were briefly stained with DAPI to reveal the cell nuclei. After washing with DPBS, fluorescence was visualized and photographed using an In Cell Analyzer 2000 (GE, Healthcare, Parsippany, NJ, USA) or a Leica confocal microscope. All antibodies are listed in Additional file 1: Table S6.

### Western blotting analysis

Cells in the culture plates were rinsed once with ice-cold DPBS and lysed in the RIPA buffer containing 1% PMSF and 1% protease/phosphatase inhibitor cocktail (Thermo Fisher Scientific, Waltham, MA, USA) for 30 min at 4 °C, followed by centrifugation at 12,500 g for 20 min at 4 °C. Lysates in a 1× sample buffer were boiled for 5 min at 95 °C for denaturation and separated by sodium dodecyl sulfate-polyacrylamide gel electrophoresis. The target proteins were detected by Western blotting with their respective specific antibodies, and α-Tubulin was used as an internal control. The blot was visualized using an ECL kit (GE Healthcare), according to the manufacturer’s instructions. We quantified the intensity of the bands using Image Lab 5.0 software. All antibodies are listed in Additional file 1: Table S6.

### Extracellular Aβ measurement

We measured Aβ40 and Aβ42 using cell supernatants conditioned for four days. We performed three biological replicates. Supernatants were collected at different time points, and frozen at − 80 °C. Secreted Aβ40 and Aβ42 were measured using Human/Rat β Amyloid (40) ELISA Kit (Wako) and Human/Rat β Amyloid (42) ELISA Kit (Wako, Japan), according to the manufacturer’s instructions.

### Immunoprecipitation

Immunoprecipitation of Rpl26 was performed as described previously [40]. In brief, cells were lysed in lysis buffer plus protease inhibitor and phosphatase inhibitor cocktails (Thermo Fisher Scientific, Waltham, MA, USA). Following centrifugation at 17,000 × *g*, supernatants (4 mg/ml protein) were treated with Protein A/G magnetic beads (Thermo Fisher Scientific, Waltham, MA, USA) for 2 h. Beads were removed, and supernatants were incubated with primary antibody overnight at 4 °C and then treated with magnetic beads for 2 h. After washing with lysis buffer, proteins in beads were eluted in 2× sample buffer, and analyzed using Western blotting.

### LysoTracker red staining

Cells were stained with 100 nM LysoTracker Red DND-99 (Invitrogen) for 15 min. After washed with probe-free medium, the cells were imaged using an IncuCyte® S3 Live-Cell Analysis System. The fluorescence intensity was measured by Image J. The values were normalized to the control group.

### DQ-Red BSA staining

Lysosomal-dependent proteolysis was determined by DQ-Red BSA (Molecular Probes/Invitrogen, D-12,051). The cells were incubated with DQ-Red BSA at a concentration of 10 µg/ml for 1 h (37 °C, 5% CO2) and then were washed 3 times with DPBS. After 10 h, the cells were observed using a confocal microscopy (Zeiss, LSM710, Germany). The fluorescence intensity was measured by Image J. The values were normalized to the control group.

### Statistical analysis

The statistical analysis was performed using GraphPad Prism 5.0 statistical software (GraphPad Software, Inc., San Diego, CA, USA). All experiments were performed in triplicate at least and data were expressed as mean ± standard error of the mean (SEM). Statistical analysis was carried out using one-way analysis of variance, followed by Tukey’s multiple comparison or two-sided Mann–Whitney U-test for two groups. A P value < 0.05 was considered significant.

## Results

### Generating human iPSCs with heterozygous and homozygous PS1 knock-in F105C by combining CRISPR/Cas9 and piggyBac

We established human iPSCs by reprogramming urine cells derived from a 28-year-old healthy female Chinese donor following our previously reported protocol [34]. The healthy female urine cell-derived iPSCs (UC-H2-iPSCs) displayed normal karyotypes and expressed pluripotency markers, which were confirmed by immunofluorescence staining, qRT-PCR analysis (SOX2, NANOG, ZFP42, OCT4, LIN28, and TERT), and the ability to form teratomas containing the tissues of all three germ cell layers (Additional file 1: Fig. S1). In addition, according to Using a well-defined neuronal differentiation protocol, UC-H2-iPSCs displayed the ability to efficiently differentiate into NSCs and neurons as compared with control (Additional file 1: Fig. S2).

A precise and seamless knock-in mutation is one of the biggest challenges in gene editing. The combination of CRISPR/Cas9 and piggyBac systems has been reported to successfully introduce seamless knock-in mutations in one of the bi-alleles in iPSCs [37]. However, some previous studies have suggested that it would be almost impossible to generate homozygous mutants in human iPSCs [41–43]. To knock in heterozygous and homozygous PS1 F105C mutations in UC-H2-iPSCs, we developed a modified genome editing method by combining the CRISPR/Cas9 system with two piggyBac transposons containing PuroR and HygR genes for double resistance selections (Fig. 1C). To construct a piggyBac-based donor plasmid with the PS1 F105C sequence, we synthesized an upstream genomic sequence and a downstream sequence with the PS1 F105C mutation from the TTAA site in the exon 4 of the PS1 gene. We then integrated them into the left- and right-inverted terminal repeats of the piggyBac transposon. UC-H2-iPSCs were then transfected with the donor plasmid, gRNA, and Cas9 vectors via liposome transfection. After CRISPR/Cas9-mediated targeted double-stranded breaks in the exon 4, the piggyBac transposon construct carrying the bi-functional puroR-TK gene for positive and negative selections integrated into the cleaved site via homology direct repair (HDR). Cells carrying the puroR-TK gene became resistant to puromycin and sensitive to FIAU cytotoxicity. After the selection of puromycin, we efficiently obtained heterozygous PS1 F105C mutants with an accurate HDR in 29 of the total 153 single-cell clones (18.9%) (Fig. 1D, E). However, no homozygous mutation was found in these clones.

To generate homozygous PS1 F105C mutation, we used the puromycin-selected cells with heterozygous PS1 F105C mutation for the second transfection with Cas9 vector, gRNA, and piggyBac transposon carrying the selectable markers HygR and TK. The cells were selected with hygromycin and removed piggyBac transposon sequence using the same approach. Finally, we identified seven clones with PS1 F105C homozygous mutation from 51 single-iPSC clones (13.7%) (Fig. 1E). To obtain the heterozygous and homozygous PS1 mutant without the piggyBac sequence, the colonies carrying piggyBac were transiently transfected with a transposase-expressing plasmid to remove any remnants of the piggyBac. They then were treated with FIAU to kill the rest of cells using piggyBac. To avoid single-cell clones with transposon residues, we used a series of PCRs to ensure no integration of exogenous transposon sequences (Additional file 1: Fig. S3). We used Sanger sequencing to ensure the desired mutation in the PS1 gene (Fig. 1F).

For detecting the possible off-target caused by Cas9-induced cleavage, we predicted gRNA off-targets using the COSMID tool (http://crispr.bme.gatech.edu)[38], and then amplified and sequenced the top six off-target sites for each gRNA in the corresponding clones (Additional file 1: Tables S3 and S4). Using the standard human genome as a reference sequence, we found no changes in the off-target sequence compared to the isogenic control cell. Moreover, these PS1 F105C knock-in iPSC lines retained normal karyotypes (Additional file 1: Fig. S3) and the pluripotency biomarker Oct4 (Fig. 1G). Using this seamless knock-in approach, we obtained the heterozygous PS1 mutant (PS1^+/F105C^) and homozygous PS1 mutant (PS1^F105C/F105C^) iPSCs with the same female genetic background as UC-H2-iPSCs (PS1^+/+^).

## Human PS1 F105C neurons tend to generate more Aβ

**Fig. 2:**
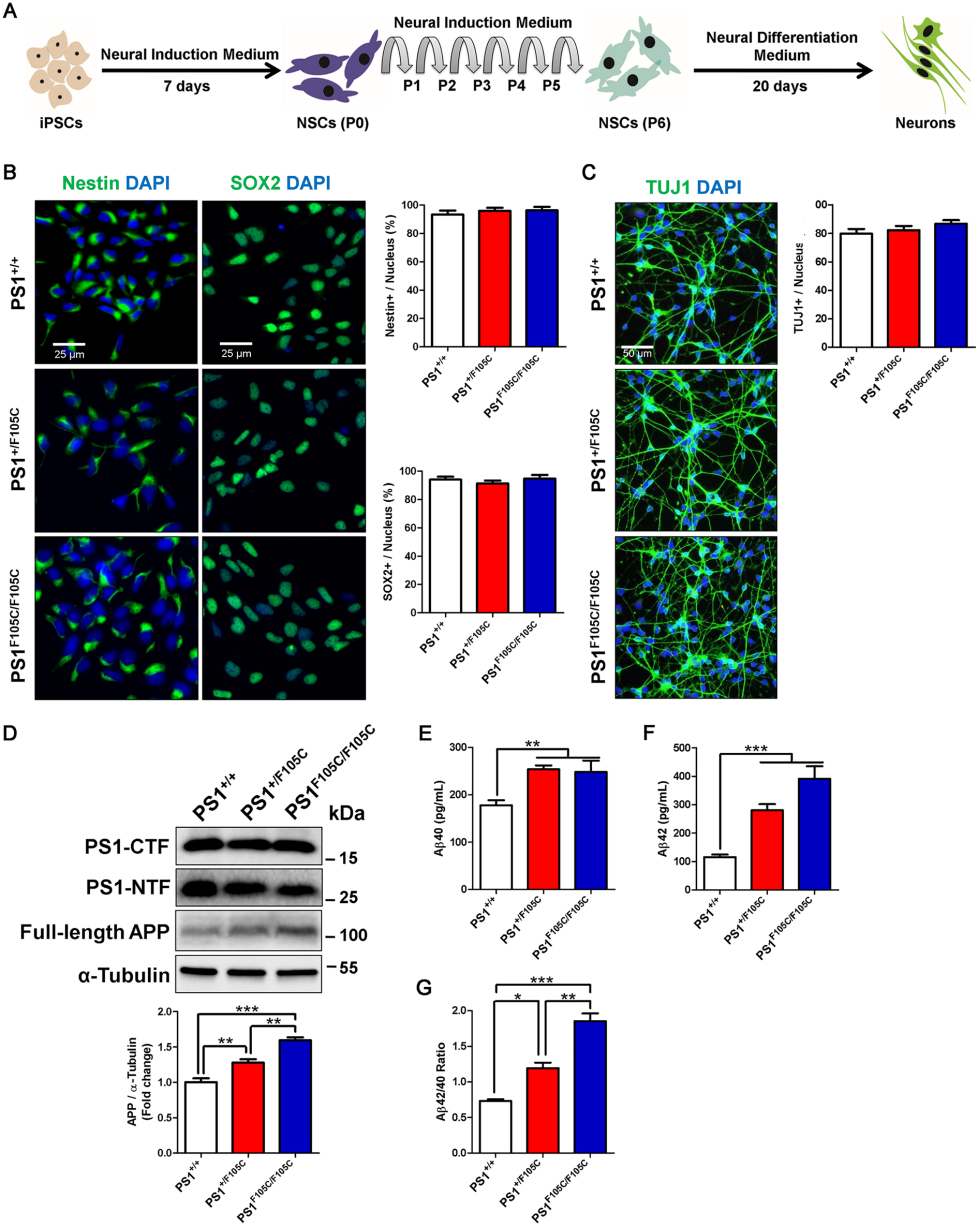
PS1 F105C mutation enhanced extracellular Aβ levels in human neurons. **A** The workflow for the neuronal differentiation of iPSCs. **B** Nestin and SOX2 immunostaining of NSCs from iPSCs and the percentage of Nestin and SOX2 positive cells (n = 3). **C** TUJ1 immunostaining of neurons from iPSCs and the percentage of TUJ1 positive cells (n = 3). **D** Representative western blotting of PS1-CTF, PS1-NTF, full-length APP, and α-Tubulin in PS1^+/+^, PS1^+/F105C^, and PS1^F105C/F105C^ neurons. Mutation dependent changes in APP in PS1 F105C neurons (n = 3). Data are represented as mean ± SEM. **P < 0.01 and ***P < 0.005 were considered significantly different. ELISA quantification of Aβ40 **E** and Aβ42 **F** secreted from neurons (n = 4). Data are represented as mean ± SEM. **P < 0.01 and ***P < 0.005 were considered significantly different. **G** Mutation dependent changes in Aβ42:40 ratios in PS1 F105C neurons. Data are represented as mean ± SEM. *P < 0.05, **P < 0.01, ***P < 0.005 were considered significantly different

Using a well-defined neuronal differentiation protocol [39], we efficiently differentiated these isogenic iPSC into NSCs for 7 days (Fig. 2A). All iPSC lines efficiently generated SOX2 and Nestin-positive cells with the same capacity (Fig. 2B). After six passages and differentiation for another 20 days, these isogenic NSCs differentiate into neurons. The immunostaining showed that these isogenic NSCs had a similar ability to differentiate into TUJ1-positive neurons (Fig. 2C). The Western blot analysis showed that the levels of endoproteolytically cleaved N-terminal and C-terminal fragments of PS1 (PS1-NTF and PS1-CTF) in PS1^+/F105C^ and PS1^F105C/F105C^ remained unchanged, compared with the PS1^+/+^ neurons (Fig. 2D), suggesting that the PS1 F105C mutation did not affect the endo-cleavage of PS1. In addition, we found that the level of full-length APP increased in mutant copy-dependent manner (Fig. 2D). ELISA assay showed that culture medium from PS1 mutant neurons had a significant increase in Aβ40, Aβ42, and Aβ42/Aβ40 ratio (Fig. 2E and G). Hence, cultured PS1 F105C mutant neurons tend to generate more Aβ similar to those in AD animal models and patients.

### mTORC1 in human PS1 F105C neurons is insensitive to starvation

**Fig. 3:**
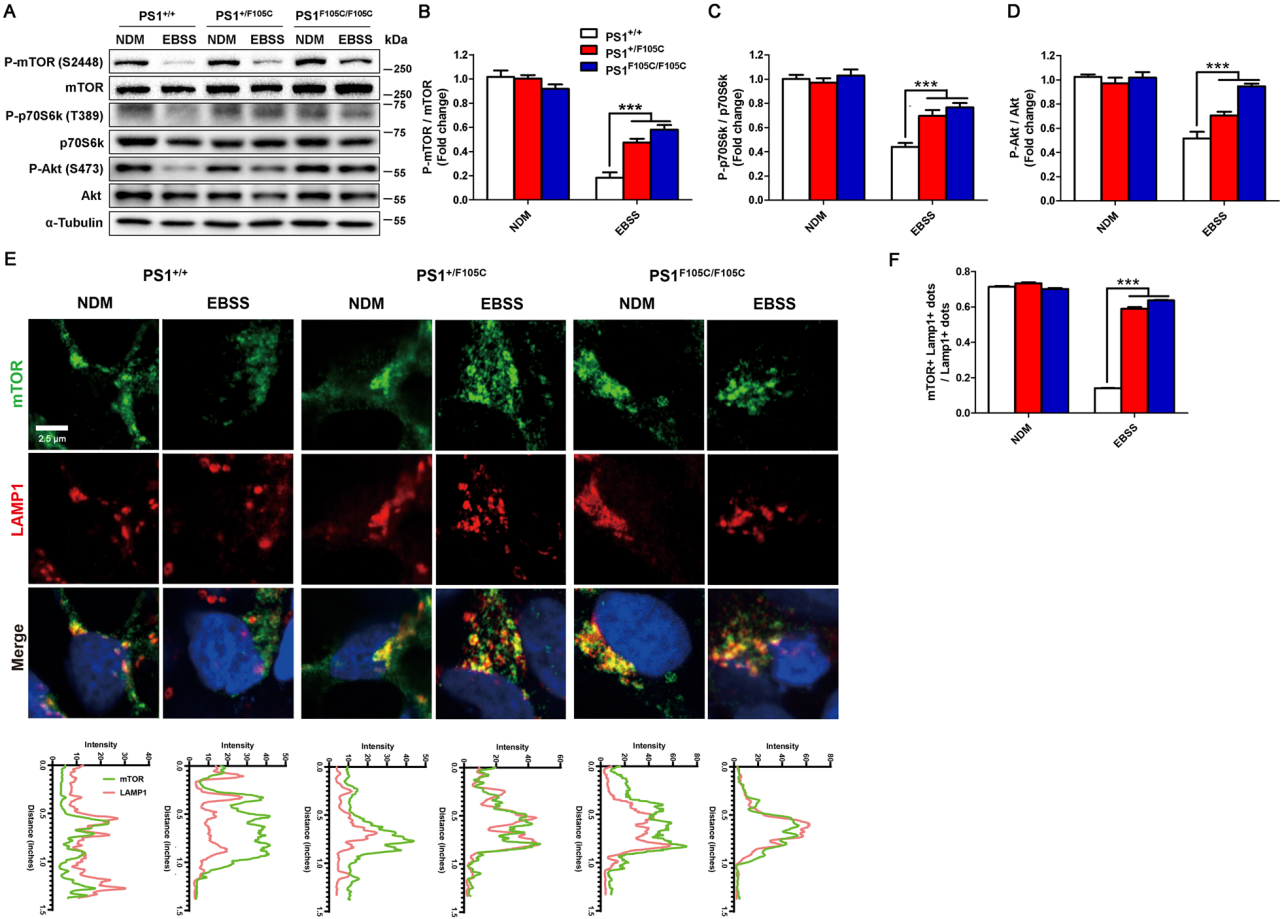
PS1 F105C mutation caused insensitive mTORC1 to starvation in human neurons. **A** Representative western blotting of p-mTOR, T-mTOR, P-p70S6k, T-p70S6k, P-Akt, T-Akt, and α-Tubulin in PS1^+/+^, PS1^+/F105C^, and PS1^F105C/F105C^ neurons after EBSS starvation for 4 h. **B** p-mTOR/T-mTOR, **C** P-p70S6k/T-p70S6k, and **D** P-Akt/T-Akt were quantified by western blotting analysis (n = 3). Data are represented as mean ± SEM. ***P < 0.005 was considered significantly different. **E**, **F** Immunofluorescence staining and image J analysis for localization of mTOR and LAMP1 after EBSS starvation in PS1^+/+^, PS1^+/F105C^, and PS1^F105C/F105C^ neurons. Co-localized dots were counted (n ≥ 30). Data are represented as mean ± SEM. ***P < 0.005 was considered significantly different

mTOR activity is sensitive to energy deprivation. Therefore, we measured the Akt/mTORC1/p70S6K pathway in these neurons under starvation (by culturing neurons in Earle’s Balanced Salt Solution (EBSS)). Western blotting analysis showed that these isogenic neurons had no difference at phosphorylated levels of mTOR (Ser2448), p70S6k (T389), and Akt (S473) in the fed condition (Fig. 3A–D) (Additional file 1: Fig. S4). Under EBSS starvation, there was a dramatic decrease in phosphorylated mTOR in isogenic control neurons, whereas phosphorylated mTOR levels still maintained higher in PS1 mutant neurons (Fig. 3A, B). PS1 F105C mutant neurons also had higher phosphorylated p70S6K levels and phosphorylated Akt levels than isogenic control neurons (Fig. 3A–D). Hence, PS1 F105C mutant neurons keep higher mTORC1 activity under starvation as determined by phosphorylated p70S6K. To verify the changes of mTORC1 activity in PS1 F105C neurons, we checked the co-localization of mTOR and lysosome marker LAMP1 (Fig. 3E, F). In isogenic control neurons, mTOR co-localized to LAMP1 positive vesicles in nutrient fed condition, whereas it diffused into the cytoplasm under EBSS starvation condition. In PS1 F105C mutant neurons, LAMP1-positive vesicles were more dispersed throughout the cytoplasm and more extensive as compared with isogenic control neurons. Under starvation, the vast majority of mTOR remained co-localization with lysosomes in PS1 mutant neurons. These results revealed that the response of mTORC1 to starvation decreases in PS1 F105C mutant neurons.

### Autophagy dysfunction in human PS1 F105C mutant neurons

**Fig. 4:**
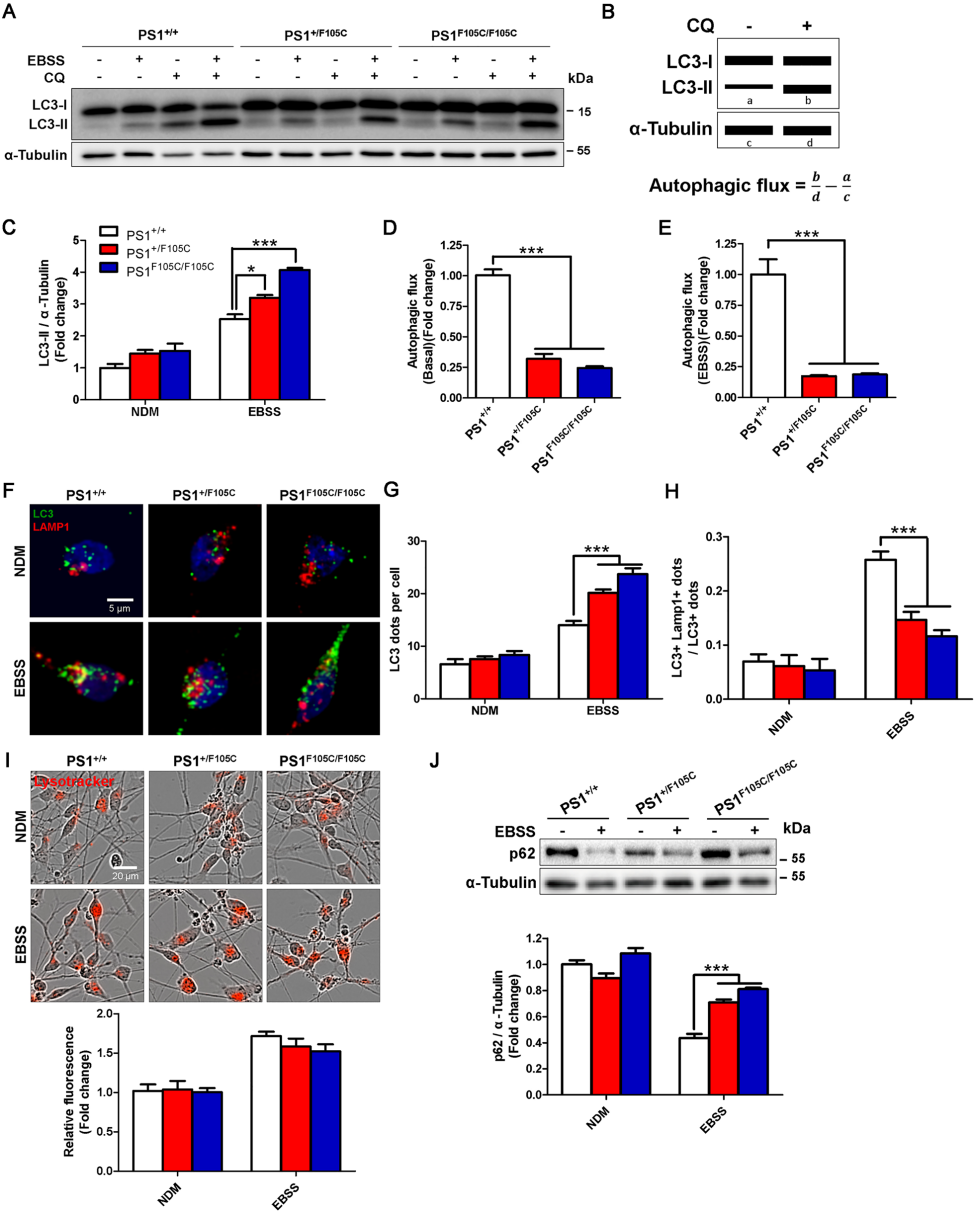
PS1 F105C mutation led to autophagy dysfunction in human neurons. **A** Representative western blotting of LC3 and α-Tubulin in PS1^+/+^, PS1^+/F105C^, and PS1^F105C/F105C^ neurons after 30 µM CQ treatment or EBSS starvation or both for 4 h. **B** Diagram of the autophagic flux assay. Autophagic flux was calculated by comparing LC3-II levels following CQ treatment. **C** LC3-II levels, **D** Basal autophagic flux, and **E** EBSS-induced autophagic flux were determined by western blotting analysis (n = 3). Data are represented as mean ± SEM. *P < 0.05 and ***P < 0.005 were considered significantly different. Data are represented as mean ± SEM. ***P < 0.005. **F** Immunofluorescence staining for localization of LC3 and LAMP1 after EBSS starvation in PS1^+/+^, PS1^+/F105C^, and PS1^F105C/F105C^ neurons. **G** LC3 dots and **H** co-localized dots were counted (n ≥ 30). Data are represented as mean ± SEM. ***P < 0.005 was considered significantly different. **I** After EBSS starvation in PS1^+/+^, PS1^+/F105C^, and PS1^F105C/F105C^ neurons, the lysosome was visualized and quantified by LysoTracker Red staining (n = 4). Data are represented as mean ± SEM. **J** The levels of p62 and α-Tubulin were detected and quantified by western blotting using specific antibodies (n = 3). Data are represented as mean ± SEM. ***P < 0.005 was considered significantly different

Since mTORC1 is a critical autophagy regulator, thus we further investigate the effects of PS1 F105C mutation on different stages of autophagy, including autophagosome formation, autophagosome-lysosome fusion, and lysosomal digestion. For autophagosome formation, we performed the western blotting analysis to test the changes of classical autophagosome marker LC3 upon induction of autophagy by EBSS-induced starvation (Fig. 4A). Chloroquine (CQ) is an autophagy-related inhibitor for blocking the fusion between autophagosomes and lysosomes. We used chloroquine (CQ) to inhibit LC3-II degradation and then monitor autophagic flux (Fig. 4B). Western blotting showed that LC3-II levels in PS1^+/+^, PS1^F105C/+^ PS1^F105C/F105C^ neurons increased after EBSS starvation for 4 h, revealing that starvation could enhance autophagosome formation in all neurons (Fig. 4C). However, we noted that LC3-II had a significant increase in PS1 mutant neurons compared with isogenic control neurons under EBSS starvation. After 30 µM CQ treatment for 4 h, we found that basal autophagic flux decreased in PS1 F105C mutant neurons compared with isogenic control neurons (Fig. 4D). Under starvation, autophagic flux in PS1 mutant neurons still was lower than in isogenic control neurons (Fig. 4E). Through immunofluorescence staining, EBSS could increase LC3 dots in isogenic control neurons (Fig. 4F, G), indicating a strengthened formation of autophagosomes under starvation conditions. However, the PS1 F105C mutant neurons had more LC3 dots than control neurons, which was consistent with western blotting.

For autophagosome-lysosome fusion, we used immunofluorescence staining to observe the distribution of LC3 and LAMP1 in these neurons under starvation (Fig. 4F, H). We found that LAMP1 co-localized to LC3 positive vesicles in PS1^+/+^ control neurons. unlike isogenic control neurons, most LC3 and LAMP1 did not have co-localization in PS1F105C mutant neurons under starvation.

For lysosomal degradation, we used bioassays to determine the lysosomal function. LysoTracker staining has been commonly used for testing EBSS-induced autophagy and the increased fluorescence intensity indicates the lower pH in the lysosome [44]. Through LysoTracker staining, we found that PS1 mutant and control neurons had identical fluorescence intensity under fed condition (Fig. 4I). In addition, an identical increase of LysoTracker staining was observed in mutant and control neurons under EBSS starvation. To test lysosomal degradation function, we used a fluorogenic substrate DQ Red BSA for monitoring lysosomal protease activity. We found that the lysosomal degradation of DQ Red BSA was identical in all isogenic neurons (Additional file 1: Fig. S5), revealing that PS1 F105C mutant neurons have the normal lysosomal degradative function. We further tested the degradation of autophagy substrates p62 under starvation. As shown in Fig. 4J, under starvation, the remaining p62 levels in PS1 mutant neurons were higher than isogenic control neurons. These results reveal that autophagosomes do not move to the lysosome well in PS1 mutant neurons, thereby leading to a decrease in the degradation of the autophagic substrate.

## The treatment with mTOR inhibitor Torin1 could clear tau accumulation in human PS1 F105C mutant neurons

**Fig. 5:**
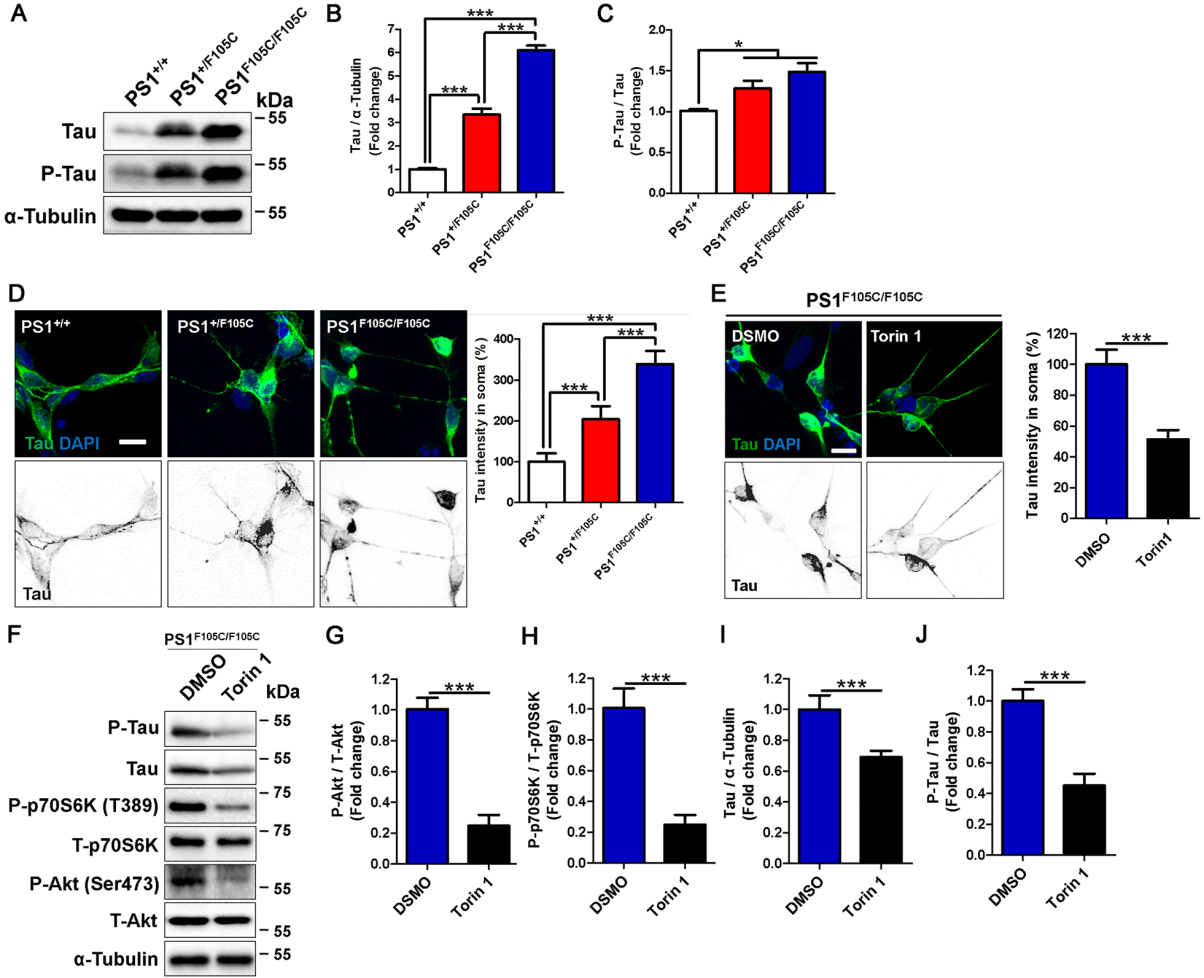
Torin 1 reduced tau phosphorylation and accumulation in human PS1 F105C mutant neurons. **A** Representative western blotting of tau, P-tau, and α-Tubulin in PS1^+/+^, PS1^+/F105C^, and PS1^F105C/F105C^ neurons. **B** Tau and **C** P-tau/tau were quantified by western blotting analysis (n = 3). Anti-tau antibody can detect all six isoforms of tau. Data are represented as mean ± SEM. *P < 0.05, and ***P < 0.005. **D** Tau immunofluorescence staining of neurons. The average intensity of tau in the soma was quantified (n ≥ 50). Neurons were treated with 1 µM Torin 1 or DMSO (vehicle control) for two days. **E** Tau immunofluorescence staining of neurons with Torin 1 or DMSO treatment. The average intensity of tau in the soma was quantified (n ≥ 50). Data are represented as mean ± SEM. ***P < 0.005 was considered significantly different. **F** Representative western blotting in PS1^+/+^, PS1^+/F105C^, and PS1^F105C/F105C^ neurons. **G** P-Akt/T-Akt, **H** P-p70S6K/T-p70S6K, **I** Tau, and **J** P-Tau/Tau were quantified by western blotting analysis (n = 3). Data are represented as mean ± SEM. ***P < 0.005 was considered significantly different

Tau pathology results from abnormally hyperphosphorylated tau protein aggregation, which positively correlates with neuronal loss and cognitive decline in AD [45–47]. Western blotting revealed that the PS1 F105C mutant neurons had a higher tau level, particularly phosphorylated tau recognized by the At8 antibody compared to isogenic control neurons (Fig. 5 A, B). Hence, the ratio of phosphorylated tau over tau was significantly increased in the PS1 F105C mutant neurons (Fig. 5A, C). Through immunofluorescence staining, we found that tau mainly distributed in the neurite of control neurons, whereas higher tau immunoreactive intensity was observed in the soma of PS1 F105C mutant neurons (Fig. 5D). As the PS1 F105C mutant copy number increased, more tau accumulated in the soma of neurons (Fig. 5D). Furthermore, the obvious fluorescence intensity of AT8 was observed in PS1 F105C neurons (Additional file 1: Fig. S6). It is unknown whether mTOR inhibition can enhance autophagy in PS1 F105C neurons, thus we test the effect of mTOR inhibitor Torin 1 on autophagic degradation of p62 in the PS1^F105C/F105C^ neurons under EBSS starvation (Additional file 1: Fig. S7). We found that Torin 1 significantly enhanced autophagic degradation of p62 in human PS1 F105C mutant neurons, suggesting that Torin 1 treatment improves the sensitivity of mTORC1 to starvation and enhances autophagic degradation in PS1 F105C mutant neurons (Additional file 1: Fig. S7). To test whether mTOR inhibition can remove tau accumulation in PS1 F105C neurons, PS1^F105C/F105C^ neurons were exposed to Torin 1 for 2 days. Immunofluorescence staining showed the effects of Torin 1 on clearing tau accumulation in PS1^F105C/F105C^ neurons (Fig. 5E). As shown in Fig. 5F–I, the western blotting analysis further confirmed that the Torin 1 treatment markedly decreased the levels of phosphorylated p70S6K, phosphorylated Akt, and tau in PS1^F105C/F105C^ neurons. Notably, Torin 1 also could reduce the hyperphosphorylation of tau (Fig. 5J). These results reveal that blocking mTOR activity efficiently cleans tau accumulation in PS1 F105C mutant neurons.

## Discussion

Due to the difficulty to obtain cells from rare FAD PS1 mutant patients, gene editing tools are commonly used for generating human iPSC with FAD PS1 mutations. A previous study reported the successful generation of isogenic iPSCs carrying the PS1△E9 mutation using TALEN-mediated gene editing [48]. However, the study did not report the efficiency for generating isogenic iPSCs carrying the PS1△E9 mutation. Recent studies demonstrated that heterozygous and homozygous PS1 knock-in mutations in human iPSCs, could be efficiently obtained by designing CRISPR/Cas9 [32, 49]. However, to screen out the desired mutations, an extremely large number of single-cell clones may be required, which is impractical for most laboratories. In this study, we first combined CRISPR/Cas9 with the piggyBac system to incorporate the mutation and resistance genes. Using double resistance screening and excision of the piggyBac sequence, we obtained iPSCs with heterozygous and homozygous PS1 mutations step by step (Fig. 1). These isogenic PS1 F105C knock-in iPSC lines retained normal karyotypes and the pluripotency biomarkers, as well as the capability to differentiate into NSCs and neurons. This suggests that the double selection system of CRISPR/Cas9/piggyBac is an effective approach to introduce heterozygous and homozygous mutations in human iPSCs. In addition, through comparison with these isogenic iPSCs-derived neurons, we demonstrated that cultured PS1 F105C mutant neurons displayed AD-related cellular characteristics such as tau hyperphosphorylation and accumulation as well as more Aβ. Notably, PS1^F105C/F105C^ neurons displayed more serious AD phenotypes than PS1^+/F105C^ neurons. Due to the interferences of wild-type PS1 in the heterozygote, the phenotype changes in PS1^+/F105C^ neurons are weaker than in PS1^F105C/F105C^ neurons. Therefore, homozygous PS1 mutant is quite important to explore the impacts of pure PS1 F105C mutation on known AD-related issues.

Deregulation of mTORC1 signaling has been observed in AD animal models and patients [50–53]. In this study, we found that mTORC1 downstream protein p70S6K (T389) exhibited higher phosphorylation in mutant neurons than in isogenic control neurons under starvation. Besides, it is known that the lysosomal localization of mTORC1 is crucial for its activity [54]. Active mTORC1 tethers to the lysosome surface, whereas autophagy induction conditions such as nutrient or growth factor deprivation can inhibit mTORC1 activity and cause the release of mTORC1 from the lysosome. We found that starvation could trigger the release of mTORC1 from lysosome membranes in control neurons, whereas mTORC1 could not release from the lysosome in starved PS1 mutant neurons (Fig. 3). We also found that mTOR phosphorylation (Ser2448) by Akt in PS1 F105C neurons was higher than in isogenic control neurons under starvation. In addition, mTORC1 upstream kinase Akt kept the activation in PS1 F105C mutant neurons under EBSS starvation. PS1 F105C mutant neurons exhibit autophagy dysfunction, such as slower autophagy flux and autophagosome accumulation. Consistently, since mTORC1 plays a critical role in negatively regulating autophagy [55], thus autophagy dysfunction in PS1F105C mutant neurons becomes more serious under starvation. In addition, it is known that mTORC2 and ribosome interaction is a conserved mechanism of mTORC2 activation [40]. The result of co-immunoprecipitation did not show any enhancement in the interaction between mTOR and ribosome protein Rpl26 under EBSS starvation (Additional file 1: Fig. S8); thus, we excluded the involvement of mTORC2. Although we provide solid evidence to support that dysregulation of Akt/mTORC1 signaling occurs in human FAD PS1 F105C neurons, it is not unclear how PS1 F105C mutation reduces the response of Akt/mTORC1 signaling to starvation.

Previous studies demonstrated that soluble tau is cleared by endosomal microautophagy and chaperon-mediated autophagy, while intracellular insoluble tau is degraded by macroautophagy [21–24]. However, hyperphosphorylated tau still accumulates in PS1 F105C neurons, revealing that autophagy system is insensitive to tau accumulation in PS1 F105C neurons. PS1 F105C mutation leads to dysregulation of Akt/mTORC1 signaling in human neurons, which maybe a critical cause for protein dyshomeostasis. Pharmaceutical blocking mTOR may bring the benefits to reducing abnormal protein accumulation. Our evidence supports this view that catalytic mTOR inhibitor Torin 1 dramatically reduced Akt phosphorylation, p70S6K phosphorylation, and tau accumulation in PS1 F105C neurons. On the other hand, abnormal mTOR activity has been considered as one of the leading events contributing to the onset and progression of AD hallmarks [56]. In the pyramidal neurons of AD patients, Akt-mediated phosphorylated mTOR has co-localization with tau accumulation in the soma [53]. The genetic modification of mTOR protein is known to affect tau synthesis and phosphorylation [53]. Our data showed that mTOR inhibition could decrease the hyperphosphorylation of tau in PS1 F105C neurons, suggesting that dysregulated Akt/mTORC1 is a critical upstream event involved in tau hyperphosphorylation and accumulation.

## Conclusion

**Fig. 6:**
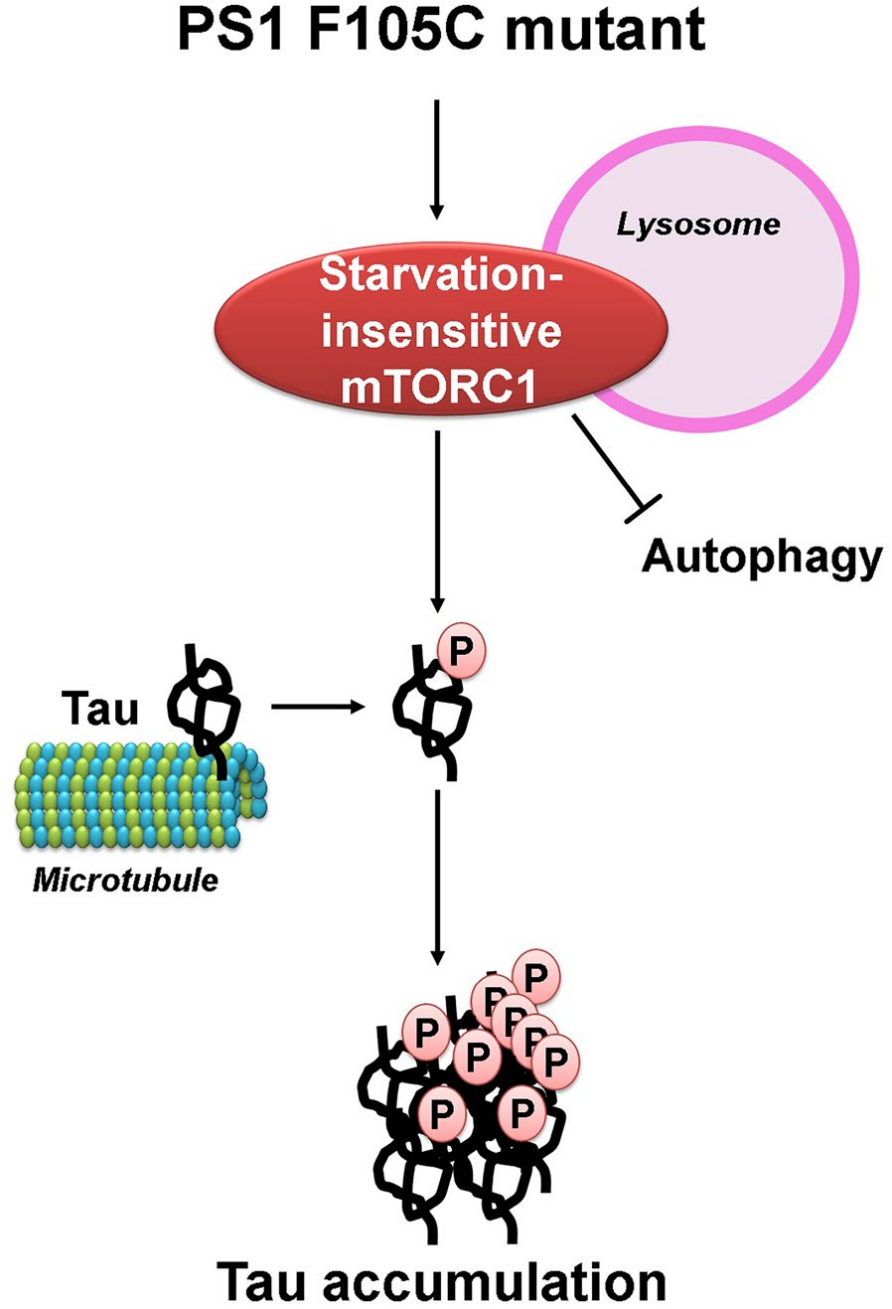
A proposed mechanism regarding how PS1 F105C mutation leads to insensitive mTORC1 signaling, contributing to human neuronal tau pathology

Our study demonstrates that PS1 F105C mutation leads to dysregulated mTORC1 signaling in human neurons, associated with tau accumulation (Fig. 6). It provides a unique perspective on the role of mTORC1 in AD-related characteristics. Given a central role in maintaining protein homeostasis, pharmaceutical blocking of mTOR may be a promising therapeutic strategy for the treatment of AD.

## Supplementary Information

**Additional file 1. **Additional tables and figures.

## Data Availability

The author declared that all and the other data supporting the findings of this study are available within the paper. The raw data that support the findings of this study are available from the corresponding author upon reasonable request.
