# Supplementary material for: Presenilin-1 F105C mutation leads to tau accumulation in human neurons via the Akt/mTORC1 signaling pathway
Source: Cell Biosci. 2022 Aug 14;12:131. doi: 10.1186/s13578-022-00874-8 (PMC9375916; doi:10.1186/s13578-022-00874-8)
Supplement: Supplementary file 1 — Additional file 1. Additional tables and figures. [file 13578_2022_874_MOESM1_ESM.docx]

**
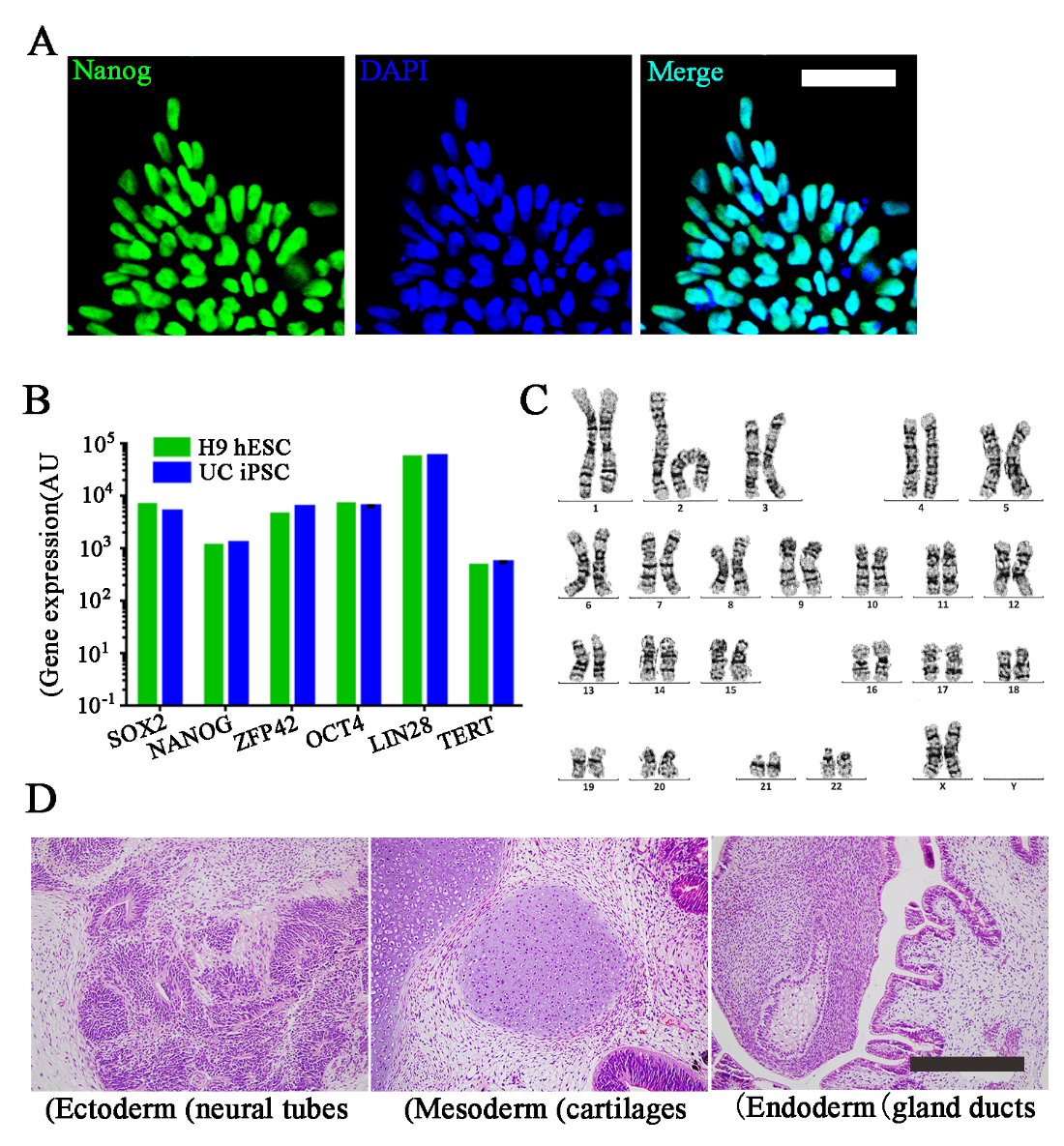
**

**Additional file 1: Figure S1.** Characterization of UC-H2-iPSCs. (A) Immunofluorescence staining of pluripotent markers of Nanog. (B) Real-time PCR analyses of pluripotent gene expression in UC-H2-iPSCs compared to H9 ESCs (n =3). (C) The UC--H2-iPSCs shows a normal human female karyotype. (D) *In vivo* teratoma derived UC-H2-iPSCs contain all three germ layers. Scale bars: 50 μm for A and 400 μm for D.


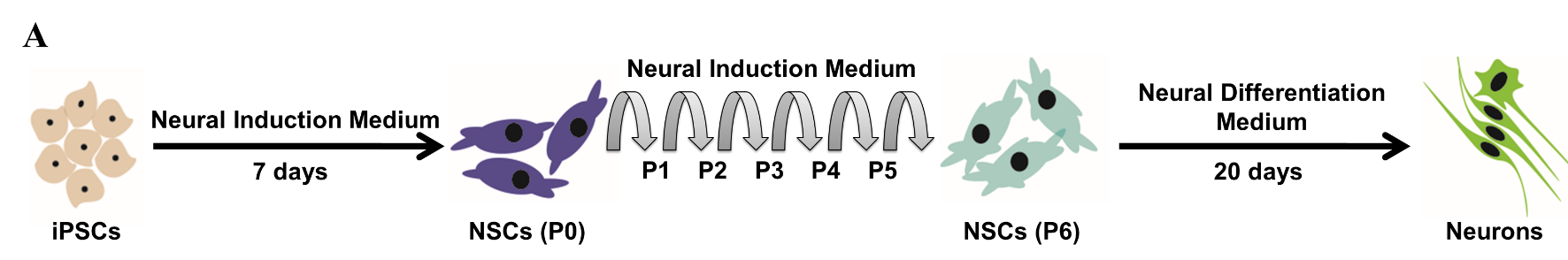
**
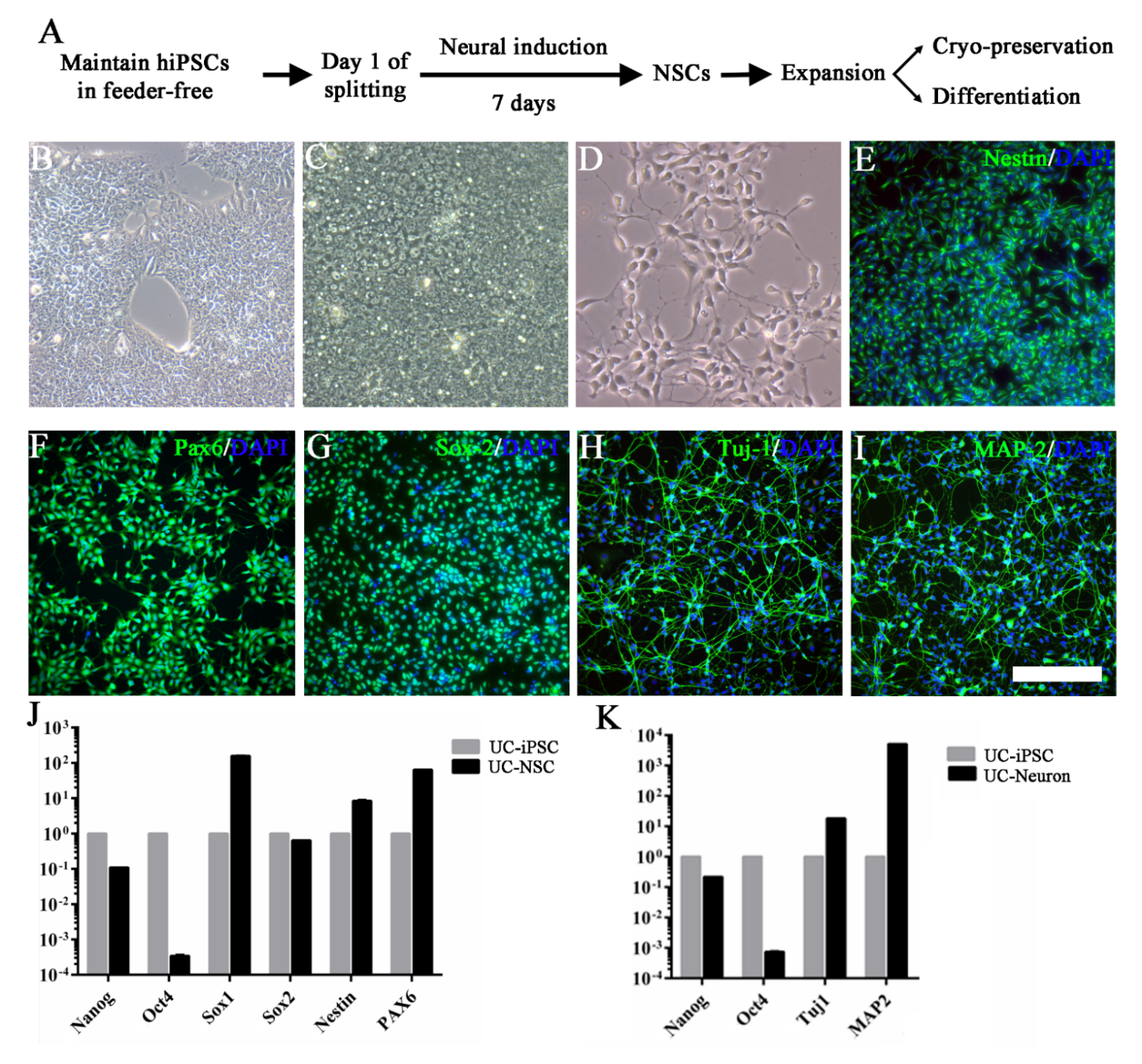
**

**Additional file 1: Figure S2.** Efficient neuronal differentiation of UC-H2-iPSCs. (A) A workflow of neural differentiation from human iPSCs. (B) Maintenance of UC-H2-iPSCs. (C) A representative phase contrast image shows that NSCs were generated after 7 days of neural induction. (D) A representative phase contrast image shows neurons were generated. Immunofluorescence staining shows the expression of specific markers for NSCs: Sox2 (E), Pax6 (F), and Nestin (G). Immunofluorescence staining of specific markers for neurons: TUJ-1 (H) and MAP-2 (I). Real-time PCR analyses of gene expression in NSCs (J) and neurons (K) compared to UC-H2-iPSCs (n=3). Scale bar: 100 μm.

**
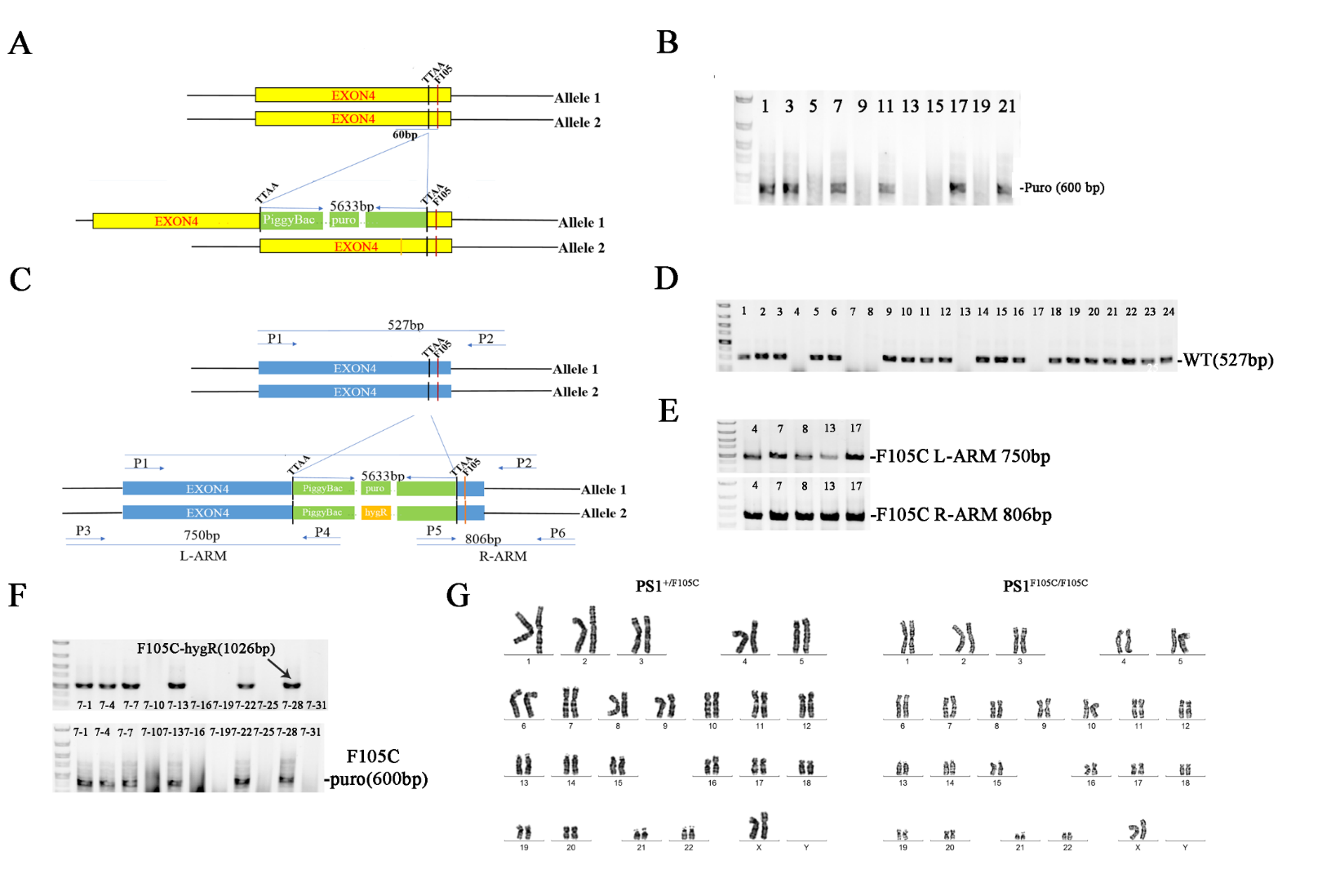
**

**Additional file 1: Figure S3.** PCR for checking PS1 F105C mutant iPSCs. (A) Schematic diagram showing piggyBac insertion site (TTAA) and intended mutated site (F105) as well as the integration of piggyBac with Puro gene into singe allele of *PSEN1*. (B) PCR for checking the Puro gene segment in *PSEN1* after puromycin resistance and FIAU selection. (C) Schematic diagram showing the integration of piggyBac with Puro and HygR gene into bi-allele of *PSEN1*, and the sites of primer pairs (D) PCR for checking the wild type *PSEN1* using primer pair (P1 and P2) after puromycin and hygromycin selection. (E) PCR for further confirming the integration of piggyBac into bi-alleles of *PSEN1* using primer pairs (P3 and P4, P5 and P6). (F) PCR for ensuring no exogenous transposon sequences integration in *PSEN1* after FIAU treatment using primers targeting puro and hygR gene segment. (G) Normal female karyotype of human iPSCs with PS1 F105C mutation.

**
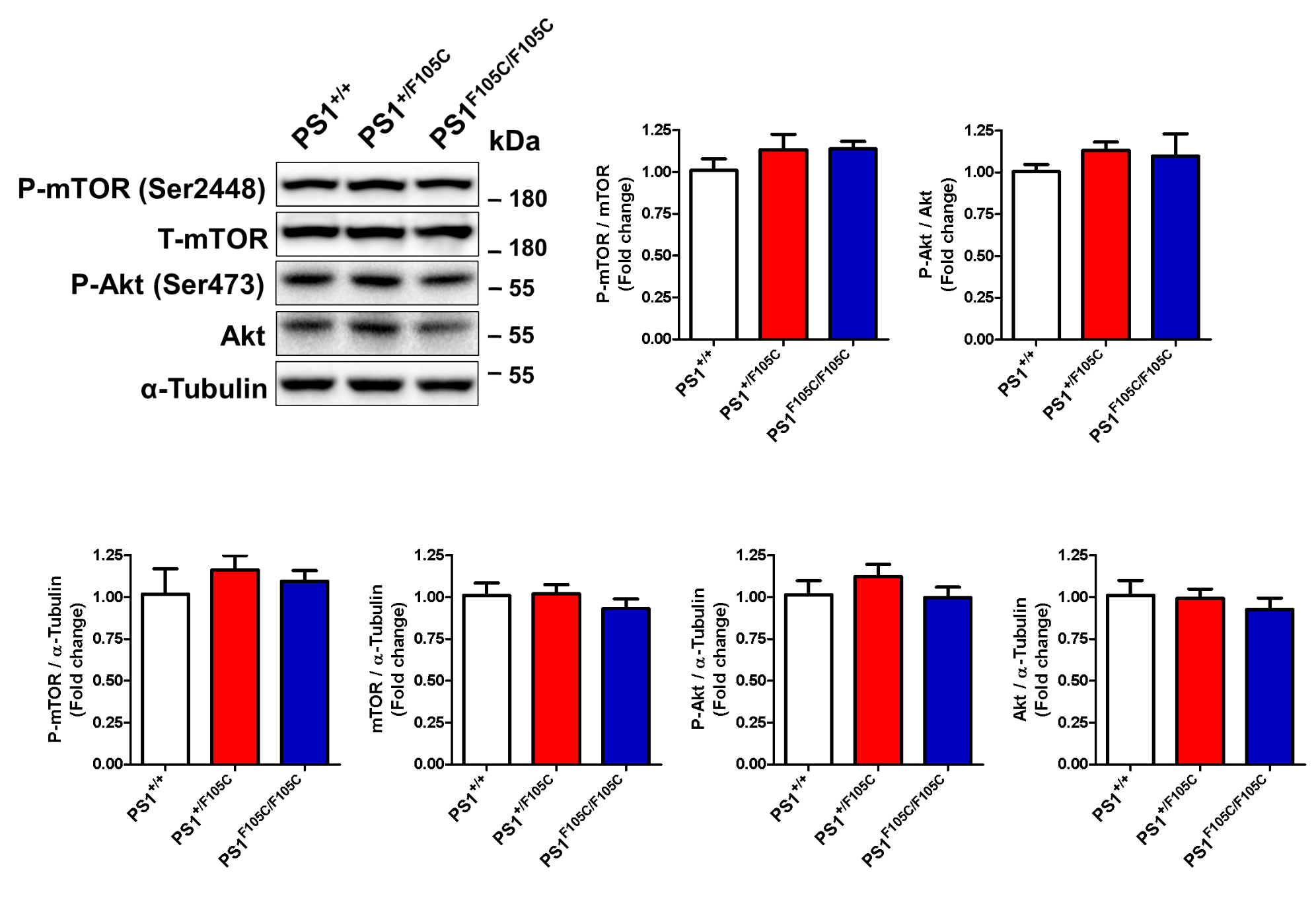
**

**Additional file 1: Figure S4.** The levels of mTOR, p-mTOR, p-Akt, and Akt in the fed condition (NDM). The bands were quantified by western blotting analysis (n = 3). Data are represented as mean ± SEM.


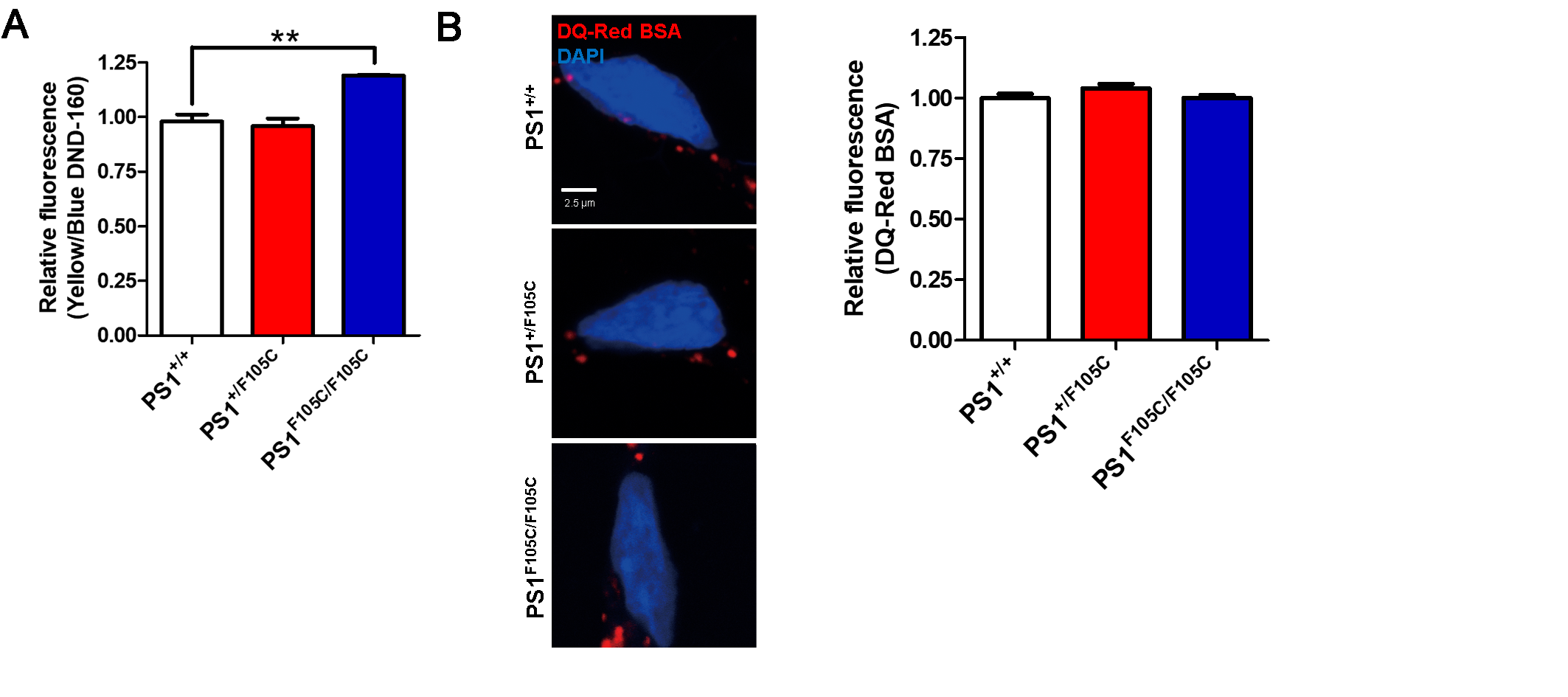


**Additional file 1: Figure S5.** Lysosomal-dependent proteolysis was visualized and quantified by DQ-Red BSA staining (n ≥ 30). Data are represented as mean ± SEM.

**
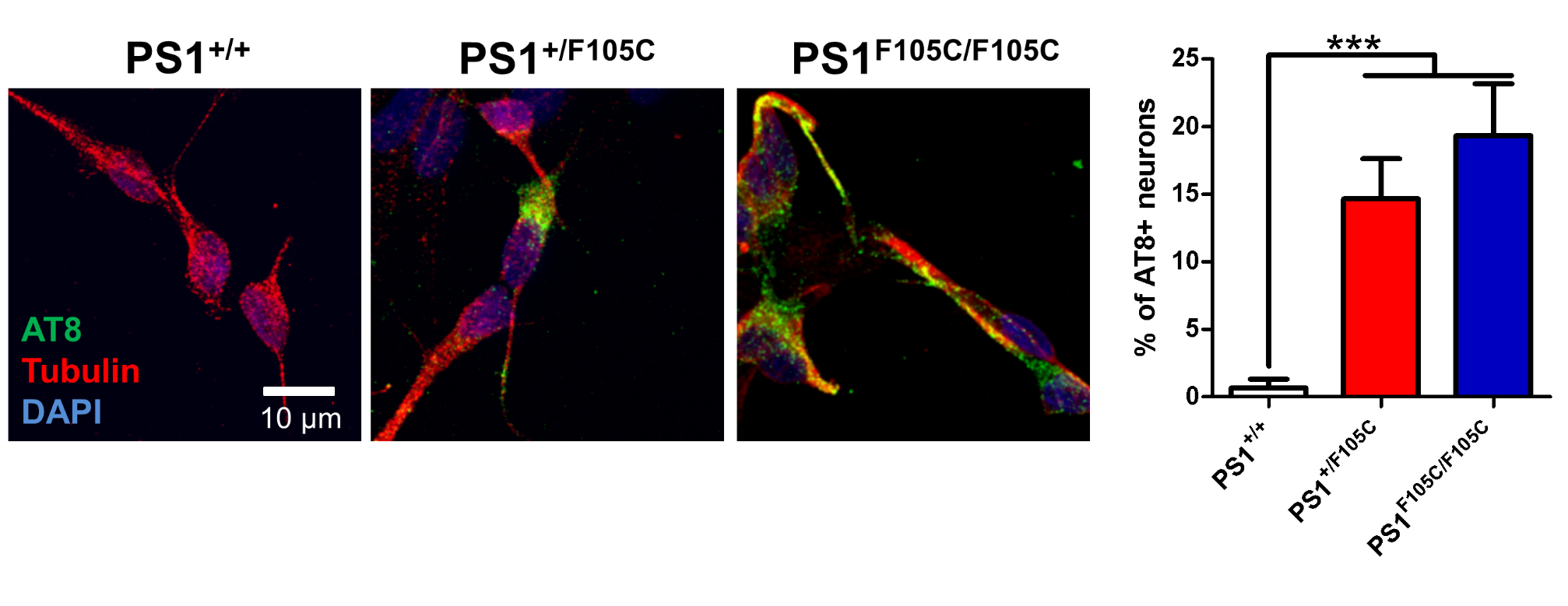
**

**Additional file 1: Figure S6.** AT8 immunofluorescence staining of neurons. The number of AT8-positive neurons was counted (n ≥ 30). Data are represented as mean ± SEM. ***P < 0.005 was considered significantly different.

**
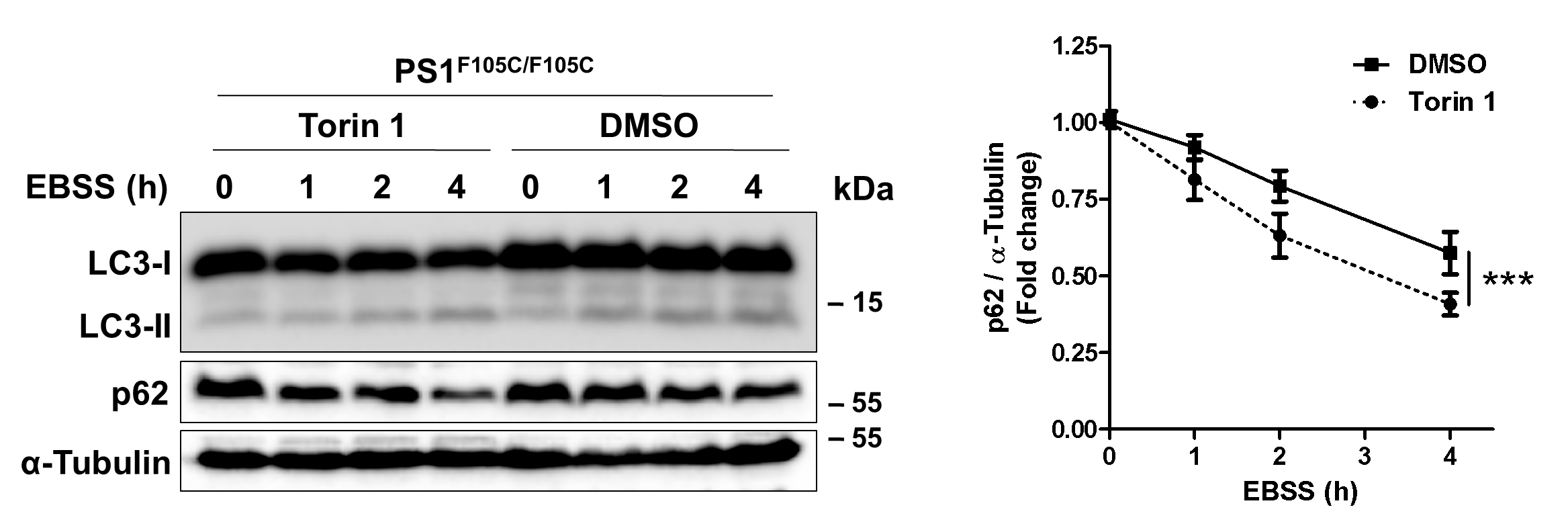
**

**Additional file 1: Figure S7.** Torin 1 enhanced autophagic degradation of p62 in human PS1 F105C mutant neurons. Neurons were treated with 1 μM Torin 1 or DMSO (vehicle control) under EBSS starvation for the indicated time. Representative western blotting of LC3, p62, and α-Tubulin in PS1^+/+^, PS1^+/F105C^. P62 / α-Tubulin was quantified by western blotting analysis (n = 3). Data are represented as mean ± SEM. ***P < 0.005 was considered significantly different.

**
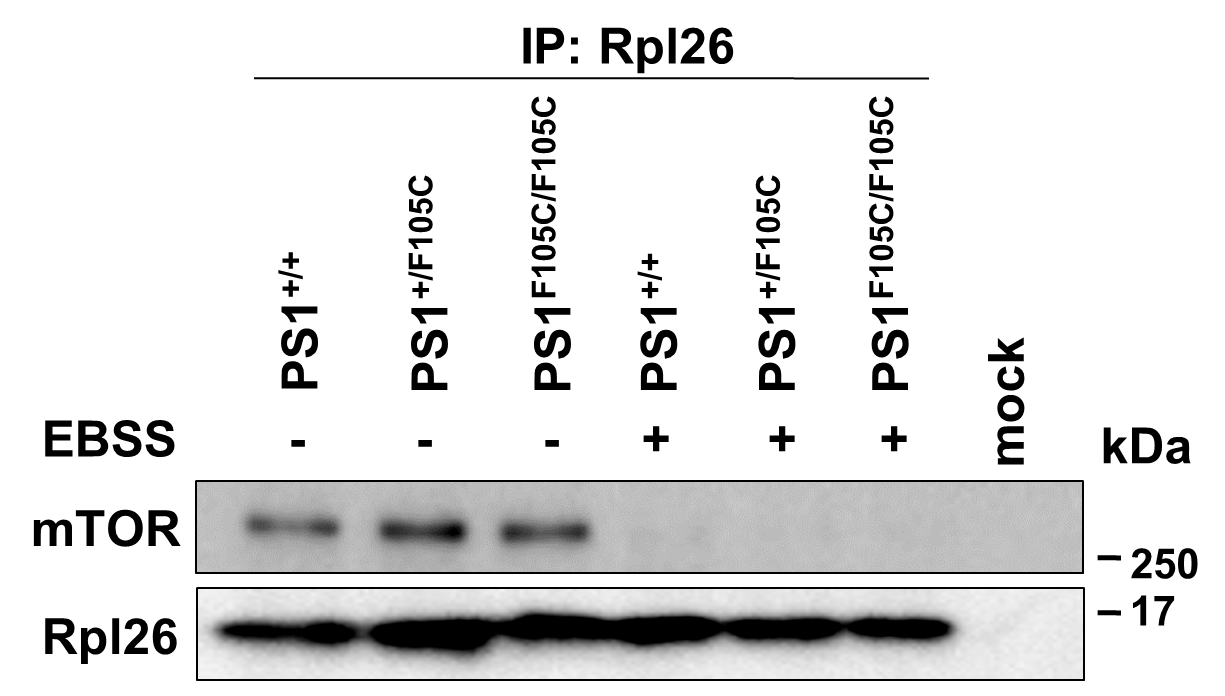
**

**Additional file 1: Figure S8.** The interaction between mTORC2 and Rp126 in neurons. The lysates from PS1^+/+^, PS1^+/F105C^, and PS1^F105C/F105C^ neurons after EBSS starvation for 4 h were immunoprecipitated with the anti-Rpl26 antibody, and obtained samples were analyzed for mTOR and Rpl26.

**Additional file 1: Tables:**

**Additional file 1: Table S1.** Primers for amplification of Puro and HygR gene.

| Primer | Sequence (5’-3’) |
| --- | --- |
| puro-seq-F | GTCGGCGGTGACGGTGAAG |
| puro-seq-R | GCTCGACATCGGCAAGGTG |
| HygR-F | ATGAAAAAGCCTGAACTCACC |
| HygR-R | TTCCTTTGCCCTCGGACGAG |

**Additional file 1: Table S2.** Primers for checking the mutation site.

| Primer | Sequence (5’-3’) |
| --- | --- |
| P1 | GGAGCACAACGACAGACGG |
| P2 | AATGGCCCTGAGGTGGAAA |
| P3 | GTCCAAACGCCTCTGACACAAGG |
| P4 | CTCACGCGGTCGTTATAGTTC |
| P5 | GTTCTTCTGATCTAGACCAATC |
| P6 | GTGTCAGGGATAAGAACC |

**Additional file 1: Table S3.** Primers for amplification of possible off-target sites

| **Primer name** | **Sequence** | **Possible off-target sites in genome** |
| --- | --- | --- |
| PS1^F105C^ -gRNA-OT7-F | GCAGGCAGGAGAGAGAGAATTTG | TACAGCACCCCATCCTTCCAGG |
| PS1^F105C^ -gRNA-OT7-R | CAACTCATGAGGGGACAGACTTTC |  |
| PS1^F105C^ -gRNA-OT8-F | GTCTCCAGCTGAGAAGAGCC | TGCAGCTGCCATCCTTCCAGG |
| PS1^F105C^ -gRNA-OT8-R | TGACCAGAGGAGGCACCACA |  |
| PS1^F105C^ -gRNA-OT9-F | AGACCACCTCCTGACACTGAGTA | TTCAGCTGCCCCTCCTTCCCGG |
| PS1^F105C^ -gRNA-OT9-R | CTAGCAGGGGCAAGTCTAACAAG |  |
| PS1^F105C^ -gRNA-OT10-F | CCCTAGATTACTCTGGAAGTGAGTCATAC | TACACCTGCCCTCCTTCCTGG |
| PS1^F105C^ -gRNA-OT10-R | CTCCTCTCTTCTTTCACTTCCTGC |  |
| PS1^F105C^ -gRNA-OT11-F | AGTCATTAAGAGTGATTGCATCAGCCATGG | TATAGCTGCCCATCCTCCAGG |
| PS1^F105C^ -gRNA-OT11-R | GGTCTTGCTTTGTCACTTGATGCC |  |
| PS1^F105C^ -gRNA-OT12-F | ATGGAAAGGACACATACGTTGGGG | TACAGCTCCCATCCCTCCAGG |
| PS1^F105C^ -gRNA-OT12-R | CACATGCTGCTATGTGTGACTGAG |  |

**Additional file 1: Table S4.** Off-target analysis on knock-in PS1^F105C^ iPSC lines. Six most possible off-target sites predicted by COSMID for PS1 F105C lines respectively were shown in the table. All off-target sites that share the same primer were considered to be one possible off-target sites. None, no off-target indels (insertion and deletion) were identified.

| **Gene** | **gRNA** | **Tool** | **ID** | **Sequence** | **Mis-match** | **Location** | **indels in het/homo** |
| --- | --- | --- | --- | --- | --- | --- | --- |
| PS1^+/+^ | 2 | COSMID | OT7 | TACAGCACCCCATCCTTCCAGG | 2 | Chr10:47094900-47094921 | none |
| PS1^+/F105C^ | 2 | COSMID | OT7 | TACAGCACCCCATCCTTCCAGG | 2 | Chr10:47094900-47094921 | none |
| PS1^F105C/F105C^ | 2 | COSMID | OT7 | TACAGCACCCCATCCTTCCAGG | 2 | Chr10:47094900-47094921 | none |
| PS1^+/+^ | 2 | COSMID | OT8 | TGCAGCTGCCATCCTTCCAGG | 1 | Chr2:26884050-26884070 | none |
| PS1^+/F105C^ | 2 | COSMID | OT8 | TGCAGCTGCCATCCTTCCAGG | 1 | Chr2:26884050-26884070 | none |
| PS1^F105C/F105C^ | 2 | COSMID | OT8 | TGCAGCTGCCATCCTTCCAGG | 1 | Chr2:26884050-26884070 | none |
| PS1^+/+^ | 2 | COSMID | OT9 | TTCAGCTGCCCCTCCTTCCCGG | 2 | Chr2:137245444-137245465 | none |
| PS1^+/F105C^ | 2 | COSMID | OT9 | TTCAGCTGCCCCTCCTTCCCGG | 2 | Chr2:137245444-137245465 | none |
| PS1^F105C/F105C^ | 2 | COSMID | OT9 | TTCAGCTGCCCCTCCTTCCCGG | 2 | Chr2:137245444-137245465 | none |
| PS1^+/+^ | 2 | COSMID | OT10 | TACACCTGCCCTCCTTCCTGG | 1 | ChrX:72371446-72371466 | none |
| PS1^+/F105C^ | 2 | COSMID | OT10 | TACACCTGCCCTCCTTCCTGG | 1 | ChrX:72371446-72371466 | none |
| PS1^F105C/F105C^ | 2 | COSMID | OT10 | TACACCTGCCCTCCTTCCTGG | 1 | ChrX:72371446-72371466 | none |
| PS1^+/+^ | 2 | COSMID | OT11 | TATAGCTGCCCATCCTCCAGG | 1 | Chr1:85250966-85250986 | none |
| PS1^+/F105C^ | 2 | COSMID | OT11 | TATAGCTGCCCATCCTCCAGG | 1 | Chr1:85250966-85250986 | none |
| PS1^F105C/F105C^ | 2 | COSMID | OT11 | TATAGCTGCCCATCCTCCAGG | 1 | Chr1:85250966-85250986 | none |
| PS1^+/+^ | 2 | COSMID | OT12 | TACAGCTCCCATCCCTCCAGG | 1 | Chr4:22064812-22064832 | none |
| PS1^+/F105C^ | 2 | COSMID | OT12 | TACAGCTCCCATCCCTCCAGG | 1 | Chr4:22064812-22064832 | none |
| PS1^F105C/F105C^ | 2 | COSMID | OT12 | TACAGCTCCCATCCCTCCAGG | 1 | Chr4:22064812-22064832 | none |

**Additional file 1: Table S5:** Real-time PCR Primers for markers of iPSCs and PS1 gene.

| **Primer** | **Sequence (5’-3’)** |
| --- | --- |
| GAPDH-qPCR-F | AGCTGAACGGGAAGCTCACT |
| GAPDH-qPCR-R | AGGTCCACCACTGACACGTTG |
| OCT4 endo qPCR-F | CCTCACTTCACTGCACTGTA |
| OCT4 endo qPCR-R | CAGGTTTTCTTTCCCTAGCT |
| NANOG qPCR-F | TGAACCTCAGCTACAAACAG |
| NANOG qPCR-R | TGGTGGTAGGAAGAGTAAAG |
| ZFP42-qPCR-F | CAGATCCTAAACAGCTCGCAGAAT |
| ZFP42-qPCR-R | GCGTACGCAAATTAAAGTCCAGA |
| LIN28-qPCR-F | GGTTCGGCTTCCTGTCCATGA |
| LIN28-qPCR-R | GGTGGCAGCTTGCATTCCTTG |
| TERT-qPCR-F | GCTTCCTCAGGAACACCAAGA |
| TERT-qPCR-R | TGCAACTTGCTCCAGACACTC |
| SOX2-qPCR-F | CCCAGCAGACTTCACATGT |
| SOX2-qPCR-R | CCTCCCATTTCCCTCGTTTT |
| SOX1-qPCR-F | TTTCCCCTCGCTTTCTCA |
| SOX1-qPCR-R | TGCAGGCTGAATTCGGTT |
| Nestin-qPCR-F | ACCCTTCCAGACTCCACTCC |
| Nestin-qPCR-R | CACACTCCTCTTCTCCCTCCT |
| PAX6-qPCR-F | TTGCTTGGGAAATCCGAG |
| PAX6-qPCR-R | TGCCCGTTCAACATCCTT |
| TUJ1-qPCR-F | gtatcccgaccgcatcat |
| TUJ1-qPCR-R | tctcatccgtgttctcca |
| MAP2-qPCR-F | GGTCACAGGGCACCTATTCA |
| MAP2-qPCR-R | TGTTCACCTTTCAGGACTGC |
| PS1-qPCR-F | TGACTCTCTGCATGGTGGTGG |
| PS1-qPCR-R | TCTCTGGCCCACAGTCTCGGT |

**Additional file 1: Table S6:** Antibodies used for Immunoprecipitation (IP), immunofluorescence (IF), and western blot (WB).

| **Antibody** | **Company & Product code** | **Concentration** |
| --- | --- | --- |
| PS1-CTF | CST, 3622 | WB, 1/1000 |
| PS1-NTF | Millipore, MAB1563 | WB, 1/500 |
| LC3 | CST, 12741 | WB, 1/1000; IF, 1/200 |
| P62 | CST, 88588 | WB, 1/1000 |
| Tau | CST, 4019 | WB, 1/1000; IF, 1/800 |
| AT8 | Thermo, MN1020 | WB, 1/1000; IF, 1/200 |
| SOX2 | Stemgent, 09-0024 | IF, 1/100 |
| Nestin | Abcam, ab22035 | IF, 1/200 |
| TUJ1 | Sigma, T8578 | IF, 1/500 |
| Nanog | Abcam, ab80892 | IF, 1/300 |
| Oct4 | Santa Cruz, sc-5279 | IF, 1/200 |
| Pax6 | Covance, PRB-278P | IF, 1/250 |
| MAP2 | CST, 4542. | IF, 1/1000 |
| mTOR | CST, 2983 | WB, 1/1000; IF, 1/200 |
| P-mTOR (Ser2448) | CST, 5536 | WB, 1/1000 |
| Lamp1 | CST, 15665 | IF, 1/100 |
| α-Tubulin | CST, 2144 | WB, 1/1000 |
| APP C-terminus | Millipore, AB5352 | WB, 1/1000 |
| Rpl26 | Bethyl Laboratories, A300685A | WB, 1/1000; IP, 2 µg/mg lysate |
